# Relationship between Antibiotic Consumption and Resistance: A Systematic Review

**DOI:** 10.1155/2024/9958678

**Published:** 2024-03-05

**Authors:** Asrat Agalu Abejew, Gizachew Yismaw Wubetu, Teferi Gedif Fenta

**Affiliations:** ^1^Department of Pharmaceutics and Social Pharmacy, School of Pharmacy, College of Health Sciences, Addis Ababa University, Addis Ababa, Ethiopia; ^2^Department of Pharmacy, College of Medicine and Health Sciences, Bahir Dar University, Bahir Dar, Ethiopia; ^3^Amhara Public Health Institute, Bahir Dar, Ethiopia

## Abstract

**Background:**

Unreserved use of antibiotics exerted selective pressure on susceptible bacteria, resulting in the survival of resistant strains. Despite this, the relationship between antibiotic resistance (ABR) and antibiotic consumption (ABC) is rarely studied. This systematic review aims to review the relationship between ABC and ABR from 2016 to 2022.

**Methods:**

Articles published over 7 years (2016–2022) were searched from December 23 to 31, 2022. The search strategy was developed by using keywords for ABC and ABR. From 3367 articles, 58 eligible articles were included in the final review.

**Results:**

The pooled ABC was 948017.9 DPDs and 4108.6 DIDs where over 70% of antibiotics were from the Watch and Reserve category based on the WHO AWaRe classification. The average pooled prevalence of ABR was 38.4%. *Enterococcus faecium* (59.4%), *A. baumannii* (52.6%), and *P. aeruginosa* (48.6%) were the most common antibiotic-resistant bacteria. Cephalosporins (76.8%), penicillin (58.3%), and aminoglycosides (52%) were commonly involved antibiotics in ABR. The positive correlation between ABR and consumption accounted for 311 (81%). The correlation between ABR *P. aeruginosa* and ABC accounted for 87 (22.7%), followed by 78 (20.3%) and 77 (20.1%) for ABR *E. coli* and *K. pneumoniae* with ABCs, respectively. Consumption of carbapenems and fluoroquinolones was most commonly correlated with resistance rates of *P. aeruginosa*, *K. pneumoniae*, *E. coli*, and *A. baumannii*.

**Conclusion:**

There is a positive correlation between ABC and the rate of ABR. The review also revealed a cross-resistance between the consumption of different antibiotics and ABR. Optimizing antibiotic therapy and reducing unnecessary ABC will prevent the emergence and spread of ABR. Thus, advocating the implementation of stewardship programs plays a pivotal role in containing ABR.

## 1. Introduction

The discovery of antibiotics is the most significant achievement in the twentieth century [1]. It changed medical practice and significantly decreased morbidity and mortality associated with bacterial infections. In recent years, the emergence and spread of antibiotic-resistant pathogens on the one hand and decreased invention of new antibiotics on the other hand challenged healthcare [2, 3]. High rates of resistance against frequently used antibiotics to treat infections have been observed worldwide, resulting in running out of effective antibiotics to treat common infections [4]. Due to this, access to antibiotics remains a critical issue globally [3, 5]. According to the Centers for Disease Control and Prevention (CDC), there were 2,868,700 infections due to resistant pathogens and 35,900 deaths from antibiotic-resistant bacterial infections each year [6].

ABR is a natural phenomenon augmented by human actions such as the inappropriate use of antibiotics [7]. Increased utilization of antibiotics results in an increased frequency of inappropriate antibiotic use [8]. Given the association between antibiotic use and the selection of resistant pathogens, inappropriate use of antibiotics is often used as a surrogate marker for the avoidable ABR [9]. Thus, the increase in bacterial resistance is contributed by selection pressure on antibiotics as a result of use, overuse, and misuse [10–12], and total consumption of antibiotics is the critical factor in selecting resistance [11]. There were individual studies that confirmed the correlation between ABC and ABR patterns [13–17]. Antibiotic stewardship programs (ASPs) are usually aimed at reducing overall ABC, and thus, preventing and reversing resistance [1, 11]. Thus, it is recommended to monitor antibiotic prescribing to improve the quality of antibiotic use and to reduce ABR [18]. Thus, this review aims to determine the relationship between ABC and rates of ABR based on articles published globally from 2016 to 2022.

## 2. Methodology

### 2.1. Study and Data Collection

**Figure 1:**
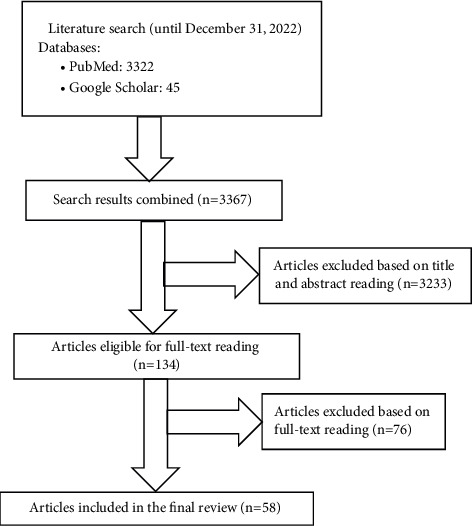
Flowchart for selection of articles for systematic review.

Eligible articles were identified by the search strategy developed by using keywords for antibiotic resistance and ABC. Mainly the PubMed (Medline) database was used to search for eligible articles. Additional articles were identified by searching from Google Scholar. Searches were performed for 7 years, and it was performed from December 23 to 31, 2022. Search terms used included a combination of keywords like “drug resistance,” “antimicrobial resistance,” “bacterial resistance,” “antibiotic resistance,” “antibiotic use,” “ABC,” “antimicrobial use,” and “antimicrobial consumption.” Articles were initially reviewed based on title and abstracts, and then, the whole document was read to select eligible articles for review. Articles that had determined the correlation between ABR and ABC using correlation coefficients and association at a *P* value of 0.05 were included in the review. Thus, articles relating to nonhuman infections, previous systematic reviews, commentaries, editorial letters, and available only in the abstracts were excluded from the review (Figure 1).

For the sake of discussion, the correlation coefficients were classified as very strong (0.9–1), strong (0.7–0.89), moderate (0.4–0.69), weak (0.1–0.39), and negligible (<0.1) [19]. Finally, the results were compiled and described. The defined daily dose (DDD)/100(0) patient days (DPDs) and DDD/1000 inhabitant days per year (DIDs) were used to report ABCs.

## 3. Results

### 3.1. General Description of Articles Included in the Review

Overall, 58 articles [20–77] published globally from 2016 to 2022 were included in the systematic review. The summary of articles included, including the correlation between bacterial resistance and ABC, is summarized and annexed in Annex 1.

The majority of the articles 13 (22.3%) were published in China [21, 25–28, 42, 60, 62–64, 68, 72, 77], 4 (6.9%) Europe [48, 49, 74, 75], 4 (6.9%) South Korea [31, 54, 61, 65], 4 (6.9%) Serbia [34, 36, 39, 52], 3 (5.2%) Japan [20, 30, 40], 2 (3.4%) Italy [22, 33], 2 (3.4%) Thailand [35, 57], 2 (3.4%) France [46, 56], 2 (3.4%) Spain [43, 59], 2 (3.4%) Slovenia [47, 71], 2 (3.4%) Switzerland [45, 70], and the rest were in different countries [23, 29, 32, 37, 38, 41, 44, 50, 51, 53, 55, 58, 66, 67, 69, 73, 76, 77]. The majority of the articles 11(23.4%) were published in 2017 [22, 33, 36, 44, 47, 48, 52, 56, 59, 64, 67], 2018 [20, 23, 26, 35, 45, 46, 53, 54, 61, 63, 65], 2019 [21, 25, 30, 31, 40, 42, 43, 50, 51, 55, 62], 2021 [24, 29, 32, 38, 43, 60, 69–71, 75, 76], and each 5 (8.6%) in 2016 [34, 37, 41, 57, 58] and 2022 [68, 72–74, 77], but only 4 were published in 2020 [27, 38, 39, 49]. Almost all 53 (91.4%) of the articles were based on retrospective data, and the rest (8.6%) were prospective observational studies.

### 3.2. Antibiotic consumption

ABC was measured by different metrics. The unit of measure for ABC was not uniform for all articles. The units of ABC were standardized to 1000 patient days (DPDs) [21–23, 25, 30, 31, 35, 37, 40, 41, 44, 46, 49–52, 55–61, 64–68, 77] and 100 patient days (DPDs) [20, 26–29, 32–34, 36, 38, 39, 42, 43, 45, 53, 69, 70, 73, 76], but in addition, DIDs [30, 40, 47, 48, 56, 59, 65, 66, 68, 71, 72, 74, 75], grams [24], and average/percentage trend [54, 62] were used to measure ABC. Accordingly, about 948017.9 DPDs (904622.9 standardized per 1000 patient days and 4339.5 per 100 patient days) and 4108.6 DIDs were reported, but about 173616.9 and 99800 were reported in average consumption and in grams, respectively. More than 70% of antibiotics were consumed in the Watch and Reserve categories based on the WHO AWaRe classification. Due to a lack of uniform measurement and recording of antibiotics among the articles, overall ABC was not summarized (Table 1).

### 3.3. Prevalence of Antibiotic Resistance

ABR was reported as a percentage (rate, trend, or prevalence) in all articles except three, which reported the incidence density of ABR [22, 52, 63, 68]. The average pooled prevalence of antibiotic-resistant bacteria was 38.4%, with *Enterococcus* faecium (59.4%), *A*. baumannii (52.6%), *P. aeruginosa* (48.5%), coagulase-negative *Staphylococcus* (43.7%), *Enterobacter* (46.1%), and *P.* mirabilis (48.5%) being the most common resistant bacteria. Cephalosporins (76.8%), penicillin (58.3%), aminoglycosides (52%), fluoroquinolones (48.3%), tetracyclines (48.6%), and carbapenems (30%) were resistant in a different way for each bacterium (Table 2).

### 3.4. Distribution of Antibiotic Consumption and Resistant Bacteria

The overall systematic review of 58 articles revealed a positive association between bacterial resistance and ABC, both in community and hospital settings. Except for some antibiotics, either increased consumption was associated with increased resistance, or decreased consumption was associated with decreased resistance. The analysis revealed that about 311 (81%) of the correlations between ABC and the ABR rate were directly related to ABC, but 73 (19%) were negatively correlated, indicating the protective nature of ABC for the development of resistance. *P. aeruginosa*, *K. pneumoniae*, *E. coli*, and *A. baumannii* were the bacteria for which a relationship between ABC and the resistance rate or pattern was commonly studied. About 73 (83.9%), 63 (81.8%), 68 (87.2%), and 53 (85.5%) antibiotics were positively correlated with the resistance rates of *P*. aeruginosa, *K*. pneumonia, *E. coli*, and *A*. baumannii, respectively (Table 3).

### 3.5. Relationship between ABC and ABR

Here, the relationship between ABC and ABR is described for each bacterium based on the correlation coefficient reported by articles [20–78]. However, it has to be noted that all studies did not provide detailed information and similar reports.

There was a very strong correlation between the consumption of meropenem and the incidence of carbapenem-resistant *P. aeruginosa* [44] and meropenem-resistant *P. aeruginosa* [69], penicillin with the incidence of combined-resistant *P. aeruginosa* [44], imipenem [57], and ertapenem [69] with imipenem resistance *P. aeruginosa* [57, 69]. There was also a very strong correlation between amikacin consumption and the resistance rate of *P. aeruginosa* to amikacin [24] and between piperacillin/tazobactam and piperacillin/tazobactam-resistant *P. aeruginosa* [69]. Similarly, there was a strong correlation between imipenem and meropenem and the rate of carbapenem-resistant *P. aeruginosa* [34], carbapenems with the rate of imipenem-resistant [44], meropenem-resistant [44], carbapenem-resistant [44], and ceftazidime-resistant [77] *P. aeruginosa*. There was also a very strong correlation between ciprofloxacin consumption and the resistance rate of *P. aeruginosa* [42].

A strong correlation was observed between the consumption of aminoglycosides and the rate of *P. aeruginosa* resistance to amikacin and gentamicin, gentamicin with the rate of aminoglycoside-resistant *P. aeruginosa* [34], and levofloxacin with the resistance rate of *P. aeruginosa* to levofloxacin [42]. A moderate correlation was found between the consumption of extended-spectrum antibiotics and cephalosporin with resistant P. aeruginosa [37], piperacillin/tazobactam with the incidence of combined-resistant *P. aeruginosa*, and amikacin with aminoglycoside-resistant *P. aeruginosa* [34]. Moderate correlations were found between the consumption of carbapenem and the rate of carbapenem-resistant *P. aeruginosa* [37, 57], carbapenem with imipenem and meropenem-resistant *P. aeruginosa* [30], and amikacin, gentamicin, and levofloxacin with resistant *P. aeruginosa* to respective antibiotics [72].

Some studies reported a decreased resistance rate of *P. aeruginosa* from the consumption of carbapenems. There was a very strong negative correlation between consumption of carbapenem and resistance rates in *P. aeruginosa* [20, 57], a strong negative correlation between carbapenem and the rate of imipenem resistance in *P. aeruginosa* [25], and a moderate negative correlation between carbapenem and the rate of carbapenem-resistant P. aeruginosa [37], and amikacin was very strongly correlated with decreased rates of multidrug-resistant (MDR) *P. aeruginosa* [57].

There was also cross-resistance between ABC and resistance to another antibiotic, which was also common. There was a very strong correlation between consumption of ceftazidime and meropenem-resistant *P. aeruginosa* [69]. There is a very strong correlation between the consumption of carbapenems and the incidence density of *P. aeruginosa* resistance to third-generation cephalosporins and aminoglycosides [22] and aminoglycosides with the rate of imipenem resistance in *P. aeruginosa* [25]. There was a very strong cross-correlation between consumption of amikacin and rates of imipenem resistance strains of *P. aeruginosa* and MDR strains of *P. aeruginosa* [57] and aminoglycosides with rates of *P. aeruginosa* resistance to tazobactam-piperacillin [39].

A strong cross-correlation was recorded between consumption of all beta-lactam antibiotics and the rate of carbapenem-resistant P. aeruginosa [34], penicillin with the resistance density of carbapenem-resistant *P. aeruginosa* [53], beta-lactam/beta-lactamase inhibitor combinations, oxacephems, sulfonamides, and quinolones with the rate of imipenem resistance in *P. aeruginosa* [25], and carbapenems with the rate of ceftazidime-resistant *P. aeruginosa* [77]. But moderate cross-resistance was observed between quinolone consumption and resistance density of carbapenem-resistant *P. aeruginosa* [53] and all beta-lactam antibiotics with rate carbapenem-resistant *P. aeruginosa* [34], carbapenems and glycoproteins with aminoglycosides-resistant *P. aeruginosa* [68], cephalosporin/beta-lactamase inhibitor and penicillin/beta-lactamase inhibitor combinations with ceftazidime-resistant P. aeruginosa [77], and penicillin/beta-lactamase inhibitor combinations, fluoroquinolones, imidazole, colistin, and tigecycline consumption with piperacillin-tazobactam-resistant *P. aeruginosa* [39].

Similar to *P. aeruginosa*, there were records on the relationship between the rate of antibiotic-resistant *A. baumannii* and ABC. Consumption of carbapenems was very strongly correlated with imipenem resistance in *A. baumannii* [25] and with carbapenem resistance in *A. baumannii* [60, 68]. Consumption of carbapenems was strongly correlated with the rate of carbapenem-resistant *A. baumannii* [33, 37, 52, 77] and carbapenems and imipenem with imipenem-resistant A. *baumannii* [55, 61], but meropenem was moderately correlated with the rate of meropenem-resistant A. *baumannii* [32].

Likewise, consumption of cefepime was very strongly correlated with cefepime-resistant *A. baumannii* [33] and tigecycline with the incidence density of *Acinetobacter* [52]. The consumption of piperacillin/tazobactam and extended-spectrum cephalosporins was strongly correlated with piperacillin/tazobactam-resistant and extended-spectrum cephalosporin-resistant *A. baumannii* retrospectively [37]. There was also a strong correlation between the consumption of fluoroquinolones and ciprofloxacin-resistant *A. baumannii* [62]. A moderate correlation was found between the consumption of cefepime and ciprofloxacin with cefepime and ciprofloxacin-resistant *A. baumannii*, respectively [32], gentamicin with gentamicin-resistant *A. baumannii* [58], and fosfomycin with the rate of fosfomycin resistance in *A. baumannii* [55]. However, there were strong negative correlations between the consumption of aminoglycosides and an incidence density of aminoglycoside-resistant *Acinetobacter* spp. [52] and a moderate negative correlation between ciprofloxacin and ciprofloxacin-resistant *A. baumannii* [37].

Cross-resistance was also reported for different antibiotics and resistant *A. baumannii*. There was a very strong correlation between consumption of ceftazidime and both imipenem and meropenem-resistant *A. baumannii* [69], cephalosporin/beta-lactamase inhibitor combinations [60, 77], and tetracyclines [60] with carbapenem-resistant *A. baumannii*. There was a strong correlation between consumption of cephalosporins, cephalosporin/beta-lactamase inhibitor combinations, and other beta-lactam/beta-lactamase inhibitor combinations with carbapenem-resistant *A. baumannii* [77], but a moderate correlation was evident between cephalosporin carbapenem-resistant *A. baumannii* [77] and glycoprotein with carbapenem-resistant A. *baumannii* [68], and monobactams were moderately correlated with rates of carbapenem-resistant *A. baumannii* [77], but amikacin was very strongly and negatively correlated with rates of MDR strain *A. baumannii*, rates of imipenem-resistant strain *A. baumannii*, and rates of resistant meropenem strain *A. baumannii* [57]. A negative strong cross-correlation was also reported between the consumption of glycopeptides and amikacin-resistant *A. baumannii* [62] and aminoglycosides, quinolones, and oxacephems with imipenem-resistant *A. baumannii* [25], whereas ciprofloxacin was strongly and negatively correlated with both imipenem and meropenem-resistant *A. baumannii* [69].

Similarly, there was a very strong correlation between the consumption of imipenem/cilastatin and the resistance rate of *K. pneumoniae* to imipenem/cilastatin and fluoroquinolones with the prevalence of ciprofloxacin-resistant *K. pneumoniae* [43], but there were very strong negative correlations between imipenem and imipenem- and meropenem-resistant *K. pneumoniae* [69]. There was a strong correlation between the consumption of carbapenems and carbapenem-resistant *K. pneumoniae* [77] and extended-spectrum cephalosporins with extended-spectrum cephalosporin-resistant *K. pneumoniae* [69]. Colistin resistance was strongly correlated with the consumption of colistin [39]. Consumption of carbapenems was moderately correlated with doripenem-resistant, ertapenem-resistant, and meropenem-resistant *K. pneumoniae* [39] and carbapenem-resistant *K. pneumoniae* [44]. Consumption of cephalosporins was also moderately correlated with the rate of cephalosporin-resistant *K. pneumoniae* [44] and fluoroquinolones with ciprofloxacin-resistant *K. pneumoniae* [61].

A very strong cross-correlation was observed between the consumption of piperacillin/tazobactam and aminoglycoside-resistant *K. pneumoniae* [22], meropenem-resistant and imipenem-resistant *K. pneumoniae* [69], fluoroquinolones with trimethoprim/sulfamethoxazole-resistant *K. pneumoniae* [22], and the beta-lactam/beta-lactamase inhibitor combinations with carbapenem-resistant *K. pneumoniae* [22]. Consumption of sulfonamides was also very strongly correlated with the rate of ceftazidime-resistant *K. pneumoniae* and ceftazidime with quinolone-resistant *K. pneumoniae* [25]. There was a strong correlation between colistin-resistant *K. pneumoniae* and imidazoles, carbapenems, and colistin [39], doripenem-resistant *K. pneumoniae* with the consumption of imidazoles, ertapenem-resistant *K. pneumoniae* with glycopeptides, and meropenem-resistant *K. pneumoniae* with glycopeptides [39], and ceftriaxone was moderately correlated with the rate of carbapenem-resistant *K. pneumoniae* [35] and carbapenems with *K. pneumoniae* resistant to third-generation cephalosporin [63]. A moderate cross-correlation was observed between the consumption of glycopeptides and carbapenem-resistant *K. pneumoniae* [68].

A very strong negative cross-resistance was found between consumption of beta-lactam/beta-lactamase inhibitor combinations with fluoroquinolone-resistant *K. pneumoniae* [22], between doripenem-resistant *K. pneumoniae* and first- and second-generation cephalosporins, and aminoglycosides [39]. There was a strong negative correlation between the consumption of ciprofloxacin, ofloxacin, and norfloxacin with carbapenem‐resistant *K. pneumoniae* [35]; colistin-resistant *K. pneumoniae* with first- and second-generation cephalosporins and penicillin/beta-lactamase inhibitor; between doripenem-resistant *K. pneumoniae* and third- and fourth-generation cephalosporin and glycopeptides; between ertapenem-resistant *K. pneumoniae* and first- and second-generation cephalosporins; and between meropenem-resistant *K. pneumoniae* and penicillin/beta-lactamase inhibitor combinations, fluoroquinolone, and tigecycline [39]. A strong correlation was also found between carbapenem and rates of ceftazidime resistance in *K. pneumoniae* [25], fourth-generation cephalosporins, and fluoroquinolone within cefepime-resistant and ciprofloxacin-resistant *K. pneumoniae* [62].

Antibiotic-resistant *E. coli* was also correlated with the consumption of several antibiotics. There was a very strong correlation between ciprofloxacin consumption with fluoroquinolone-resistant *E. coli* [65], and a strong correlation was found between fluoroquinolone and fluoroquinolone-resistant *E. coli* [48, 65], fluoroquinolones with the resistance rate of *E. coli* to levofloxacin and resistance rate of *E. coli* to ciprofloxacin [42], and ciprofloxacin with ciprofloxacin-resistant *E. coli* [37], but there was a strong negative correlation between fluoroquinolone and ciprofloxacin-resistant *E. coli* [62]. There was also a very strong correlation between cefotaxime and cefotaxime-resistant *E. coli* and a strong correlation between cefoxitin and cefoxitin-resistant *E. coli* [65], gentamicin with resistant *E. coli* [33], extended-spectrum cephalosporin with resistant *E. coli* [37], and imipenem/cilastatin with imipenem/cilastatin-resistant *E. coli* [62].

There was a very strong cross-correlation between third- and fourth-generation cephalosporin use and *E. coli* resistance rates to cefotaxime and cephamycin use with *E. coli* resistance rates to cefoxitin [65]. A strong cross-correlation was found between carbapenems and third-generation cephalosporin-resistant *E. coli* [61] and beta-lactam/beta-lactamase inhibitor combinations with resistance density of third-generation cephalosporin-resistant *E. coli* [62], but fourth-generation cephalosporins were negatively correlated with cefepime-resistant *E.*coli, glycopeptides with amikacin-resistant *E. coli* [62], aminoglycosides [35, 61], and ofloxacin with carbapenem-resistant *E. coli* [35].

Antibiotic-resistant *N. gonorrhoeae* was reported in two studies [23, 49]. Consumption of ceftriaxone was strongly correlated with resistant *N. gonorrhoeae*, but consumption of ciprofloxacin was moderately correlated [23]. Macrolide consumption and cefixime resistance in *N. gonorrhoeae* and consumption of cephalosporin, macrolide, and quinolone were strongly correlated with ciprofloxacin resistance in *N. gonorrhoeae* [49]. Consumption of quinolones and cefotaxime was positively associated with ciprofloxacin-resistant and cefotaxime-resistant *N. gonorrhoeae*, respectively [74].

Consumption of aminoglycosides, quinolones, and carbapenem was strongly correlated with amikacin-resistant *E. cloacae* [25]. But carbapenem-resistant *E. cloacae* showed significant, very strong negative correlations with the usage of penicillin/beta-lactamase inhibitor I combinations, beta-lactam/beta-lactamase inhibitor combinations, meropenem, and carbapenems, but a very strong positive correlation was shown with total cephalosporin use [60]. Consumption of piperacillin/tazobactam was very strongly correlated with resistant *C. difficile*, but it was very strongly but negatively correlated with vancomycin [31] and ESBL-positive *Enterobacteriaceae* rates [33]. A positive association was also reported from the consumption of macrolides and clarithromycin-resistant *H. pylori* [50, 75] and consumption of quinolones with levofloxacin-resistant *H. pylori* [75].

Consumption of meropenem was very strongly correlated with piperacillin/tazobactam-resistant *Enterobacter* spp. [69] and meropenem and ciprofloxacin with piperacillin/tazobactam-resistant *Enterobacter* spp. [69], and piperacillin/tazobactam and resistant *C. difficile* [31] and strong correlation between carbapenems with carbapenem-resistant *E. cloacae* [77], aminoglycoside and quinolone with amikacin-resistant *E. cloacae* [25], and fluoroquinolone and MDR *H. influenzae* and *Shigella* spp. [41], but the moderate correlation was shown between macrolide and macrolide-resistant *T. pallidum* [51], carbapenem and carbapenem-resistant *Enterobacteriales* [70], and carbapenems, monobactams, cephalosporin/beta-lactamase inhibitor, and penicillin/beta-lactamase inhibitor with carbapenem-resistant *E. cloacae* [77]. A very strong negative correlation was reported between the consumption of vancomycin and resistant *C. difficile* [31] and piperacillin/tazobactam and third- and fourth-generation cephalosporin-resistant *P. mirabilis* [22]; a strong negative relationship was observed between piperacillin/tazobactam and piperacillin/tazobactam-resistant *Proteus* spp. and ESBL-positive *Enterobacteriaceae* [33].

A very strong correlation was observed between the consumption of beta-lactams or beta-lactam/beta-lactamase inhibitors and methicillin-resistant *S. aureus* [22] and clindamycin with oxacillin-resistant *S. aureus* [69]. There was a strong correlation between the consumption of extended-spectrum cephalosporin [70] and cloxacillin [48] and methicillin-resistant *S. aureus* [48, 70], and third-generation cephalosporins, carbapenems, glycopeptides, and monobactams were also moderately correlated with MRSA [63]; monobactams and sulphonamides were also were moderately correlated with MRSA [64]. A very strong correlation was between the consumption of linezolid [38] and vancomycin [69] with vancomycin-resistant *E. faecium* [38, 69]. A moderate correlation was shown between glycopeptides and glycopeptide-resistant *E. faecalis/faecium* [70], penicillin with penicillin resistance in *S. pneumoniae* [71], and extended-spectrum penicillin and penicillin/beta-lactamase inhibitor combinations with the resistance of *S. pneumoniae* to respective antibiotics [71].

There was a very strong negative correlation between the consumption of fluoroquinolone with ciprofloxacin-resistant *E. faecalis* [62] and third-generation cephalosporins and Hlr-gentamicin-resistant *E. faecalis* [22] and strong negative correlation between the consumption of fluoroquinolone and ciprofloxacin-resistant *S. aureus*, glycopeptides, and gentamicin-resistant *S. aureus*, glycopeptides, and gentamicin-resistant CoN *Staphylococcus*; glycopeptides with amikacin-resistant *E. faecium* and gentamicin-resistant *E. faecalis* [62] and piperacillin/tazobactam and vancomycin with oxacillin-resistant *S. aureus* [33]. A moderate correlation was shown between the consumption of gentamicin and gentamicin-resistant *E. faecalis* [48] and fluoroquinolone with ciprofloxacin-resistant *CoN Staphylococcus* [62]. The consumption of amoxicillin/clavulanate and oxacillin resistance in *S. aureus* [33] and imipenem/cilastatin and imipenem/cilastatin-resistant MRSA were very strongly and negatively correlated [42]. Consumption of glycopeptides and aminoglycosides was negatively correlated with MRSA [64].

## 4. Discussion

ABR is identified as one of the top threats to public health and challenges progress in health care, food production, and life expectancy [6, 78]. Due to overuse and misuse of antibiotics, bacteria have become increasingly resistant to various antibiotics [1]. A recent point prevalence survey at the global level reported about 136 million per year hospital-associated infections resistant to antibiotics caused by high-priority pathogens (*E. coli, Acinetobacter* spp.*, Klebsiella* spp.*, S. aureus, Enterobacter* spp.*, and Pseudomonas* spp.) [79]. The current review revealed 38.4% of the average pooled prevalence of antibiotic resistance to bacteria, with *E.* faecium (59.4%), *A. baumannii* (52.6%), *P. aeruginosa* (48.5%), coagulase-negative *Staphylococcus* (43.7%), *Enterobacter* (46.1%), and *P. mirabilis* (48.5%). This complements the global concerns on the ABR [1, 6, 78, 79]. The burden of the *ESKAPE* and antibiotic resistance pattern calls for surveillance of antibiotic resistance targeting these pathogens by incorporating them into the infection control policy [80, 81]. It requires preventing infections as priority, slowing the development of ABR through better antibiotic use, and stopping the spread of ABR when it develops [78].

According to a recent report from a global point prevalence survey, high use of antibiotics (62.0%) and prolonged surgical antibiotic prophylaxis (60.9%) were the most common problems [82]. Thus, overuse and inappropriate use of antibiotics are recognized as a significant driver of the emergence of resistant strains of bacteria as the increased exposure to antibiotics encourages the development of ABR [83–85]. Increased ABC of broad-spectrum and last-resort antibiotics is a serious concern for public health [84–86]. Inappropriate consumption of antibiotics has also been shown to contribute to the occurrence of MDR organisms [86]. The current review [20–78] has clearly shown the relationship between ABC and ABR. It revealed more than 70% consumption of antibiotics from the Watch and Reserve categories based on the WHO AWaRe classification, among which consumption of carbapenems and fluoroquinolones were the most frequently involved in ABR including cross-resistance with consumption of other antibiotics. This was in line with a recent report from the global level which concluded, “*a large proportion of prescriptions for key Watch antibiotics were issued for indications other than those for which they were included in the Essential Medicine List*” [82]. Thus, a direct epidemiological relationship between ABC and the emergence and dissemination of resistant bacterial strains needs to be worked on for further understanding.

Biofilm-forming pathogens such as *P. aeruginosa, A. baumannii, C. jejuni, C. difficile, C. perfringens, E. faecalis, K. pneumonia, P. mirabilis, B. cepacia, P. pseudomallei, H. influenza, E. coli, S. aureus, S. epidermidis, S. pneumonia, S. pyogenes, Salmonella* spp.*, L. monocytogenes, S. agalactiae, and V. cholerae* are very difficult to treat with conventional antibiotics due to their greater resistance profile [87, 88]. The CDC and WHO classified most of these microorganisms as threats to healthcare [6, 78] and call for close monitoring of resistance patterns for highly susceptible antibiotics [6, 78]. WHO classified carbapenem- and third-generation cephalosporin-resistant *P. aeruginosa*, *A. baumannii*, *Enterobacteriaceae*, *K. pneumoniae*, *E. coli*, *Enterobacter* spp., *Serratia* spp., *Proteus* spp., *Providencia* spp., and *Morganella* spp. as critical threats (top priority) to health care and vancomycin-resistant *E. faecium*, methicillin-resistant and vancomycin-intermediate and resistant *S. aureus*, clarithromycin-resistant *H. pylori*, fluoroquinolone-resistant *Campylobacter* spp., fluoroquinolone-resistant *Salmonella* spp., and third-generation cephalosporin-resistant fluoroquinolone-resistant *N. gonorrhoeae* as a high priority [78]. The direct relationship between ABCs as evidenced from the current review [20–78] indicates a need for developing new therapeutic strategies that can be effective against common infections in addition to optimizing the use of available antibiotics.

Similarly, the CDC classified carbapenem-resistant *Acinetobacter*, *C. difficile*, carbapenem-resistant *Enterobacteriaceae*, and drug-resistant *N. gonorrhoeae* as urgent threats and drug-resistant *Campylobacter*, extended-spectrum beta-lactamase-producing *Enterobacteriaceae*, vancomycin-resistant *Enterococci*, MDR *P. aeruginosa*, drug-resistant nontyphoidal *Salmonella*, drug-resistant *Salmonella serotype Typhi*, drug-resistant *Shigella*, methicillin-resistant *S. aureus*, drug-resistant *S. pneumoniae*, and drug-resistant *Tuberculosis* as serious threats to health care [6]. As can be noted, the current review strengthens the need to approach pathogens as a major issue problem related to resistance against antimicrobial agents.

Antibiotics with “high resistance potential,” even with limited use, have been associated with the emergence of MDR Gram-negative bacteria, e.g., imipenem, ceftazidime, gentamicin/tobramycin, and ciprofloxacin [89]. ASP is known to result in decreased ABC and ABR [90]. Due to a strong association between ABC and the development of antibiotic resistance [83], reducing the need for and inappropriate consumption of antibiotics through the implementation of appropriate ASPs can help delay the emergence and spread of ABR [90, 91]. Prudent use of antibiotics has to be a pillar in the fight against ABR [82]. This requires aligning practice with evidence which requires changes in prescribing behavior based on the implementation of effective strategies to modify prescribing practices by aligning them with evidence-based recommendations for diagnosis and management [92]. Thus, the initial step in fight against ABR in healthcare institutions has to be to measure the situation of antimicrobial use and resistance in their setting to raise awareness on areas of improvement of local prescribing behaviors [82]. The current review will help to understand the relationship between ABC and resistance and justify the need for universal funding and urgency for implementation of ASPs taking into account the local context for sustainable behavioral change in physicians' antibiotic prescribing practices.

The review will serve as an important basis to better understand the issue and will inform policies, regulations, and interventions to optimize antibiotic use while reducing ABR. However, it has to be noted that the review does have limitations. The review includes literature in the English language and was not evaluated by independent evaluators. Because of the multiple numbers of antibiotics and microorganisms tested, risk assessment for bias and quality was not considered. Since most of the studies were done retrospectively, they may not necessarily show the true relationship between ABC and the rate of resistance. There are no data on the relationship between the consumption of antibiotics and the respective resistance rates in all parts of the world.

In conclusion, there was a strong relationship between antibiotic consumption and antibiotic resistance. The review also revealed a significant cross-resistance among different antibiotics. Most correlations were positively related, but a minority was negatively correlated, indicating the protective nature of antibiotic consumption for the development of resistance. There was a very strong correlation between fluoroquinolones, carbapenems, aminoglycosides, and penicillin consumption and respective resistance rates, but colistin, imipenem/cilastatin, and fluoroquinolones consumption was strongly correlated with the resistance rate of *K. pneumoniae*. Carbapenems were the most commonly used and strongly correlated with the rate of resistance for *A. baumannii*. Strict use of antibiotics is thus crucial to minimize the risk of emerging resistant organisms. ASP will help to optimize antibiotic therapy while reducing the amount of ABC to prevent the development and rate of ABR. Systems to assess institutional antibiotic utilization and the relationship between the trend of antibiotic use and rates of antibiotic resistance are strongly needed in health facilities to reduce resistance. Furthermore, special emphasis should be paid to antibiotics in the Watch and Reserve category of the WHO AWaRe classifications such as carbapenems, fluoroquinolones, macrolides, glycopeptides, and second-fourth generation cephalosporins.

## Figures and Tables

**Table 1:** Pooled antibiotic consumption by different metrics of measurement, 2016–2022 (*N* = 58).

Antibiotics	Metrics				
	Average	DDD/100 patient days	DDD/1000 patient days	DDD/1000 inhabitant days	Grams
Amikacin		6.0	22094.2	20.9	3300
Aminoglycosides	155.2	12.4	797.6		
Aminopenicillins				944.0	
Aminopenicillins and enzyme inhibitors				682.3	
Amoxicillin		1.5	110.9		
Amoxicillin/ampicillin		2.7			
Amoxicillin/clavulanate		104.4	258.0		
Amphenicols			5.2		
Ampicillin			21.4		
Ampicillin/sulbactam		3.0	61.3		
Aminoglycosides		3.8			
Antipseudomonal			68.9		
Benzylpenicillin			2.2		
Biapenem		154.6	7.1		
Carbapenems	548.2	190.9	888.0	2.2	
Cefazolin		2			
Cefepime		8.6	8382.6	6.1	
Cefixime			72.0		
Cefotaxime			2156.2	0.1	
Cefoxitin				0.0	
Cefpodoxime			38.0		
Ceftazidime		3.5	70434.2	2.9	
Ceftazidime/cefepime		3.2			
Ceftriaxone		136.3	128296.9		2500
Cefuroxime		5.0	42.1		
Cephalosporins	53411.4	256.1	14660.7	658.8	
Cephamycin			2200.0	0.1	
Ciprofloxacin		136.6	275875.2	26.7	3000
Clarithromycin			10.7		
Clindamycin		6.3	53.4		1000
Cloxacillin/cefazolin			85.2		
Colistin		7.1	883.3		
Daptomycin			6.24		
Doripenem			2.53		
Doripenem		72.5			
Ertapenem		4.1	79.8		
Erythromycin			0.8		
Flucloxacillin		5			
Fluoroquinolones	28630.6	58.7	7554.0	632.1	
Fosfomycin			5.4	0.0	
Gentamicin		81.4	81311.8	42.7	
Glycopeptides	298.3	11.1	118.6		
Imipenem		225.7	57558.2		
Imipenem/cilastatin			99.2	2.6	
Levofloxacin		100.1	573.4	7.1	
Lincosamides			44.5		
Linezolid	21504.6	365.9	5.3		
Macrolides		2.2	9552.3	102.55	
Meropenem		607.3	50276.5	6.3	4800
Metronidazole		102.5	85.8		
Monobactams			4.4		
Moxifloxacin		1.91	153.3		
Nitrofurantoin			0.0	1.32	6000
Norfloxacin		0.8	66.5		
Ofloxacin			141.5		
Oxacephems			30.0		
Oxacillin		2	13.4		6000
Oxazolidinone			7.5		
Panipenem		30.5			
Penicillin		54.9	308.5	12.5	
Piperacillin/tazobactam	69068.6	77.2	8614.4	22.3	65000
Polymyxin			2.9		
Rifampicin			84.2		
Sodium fusidate			0.3		
Sulfonamides			30.0		
Teicoplanin		1.6	38.9		
Tetracyclines			53.5	890.2	
Tigecycline		8.5	27.0		
Tobramycin			4.7		
Trimethoprim/sulfamethoxazole		1.7	156049.4	0.2	2200
Vancomycin		214.4	97.0		6000
Others		1265.7	4109.8	44.8	0
Total	173616.9	4339.5	904622.9	4108.6	99800

**Table 2:** Average pooled prevalence of antibiotic resistance, 2016–2022 (*N* = 58).

Antibiotics	*A. baumannii*	CON *staphylococci*	*E. coli*	*Enterobacter*	*Enterobacter cloacae*	*Enterobacteriaceae*	*Enterococcus faecalis*	*Enterococcus faecium*	*H. influenza*	*H. pylori*	*K. pneumoniae*	*N. meningitidis*	*P. aeruginosa*	*P. mirabilis*	*S. aureus*	*S. pneumonia*	Total
3GC			24.4					60			37.5						40.6
Amikacin	43.3		1.8		17.8	5.3					9.4		17.7				16.6
Aminoglycosides	60.8		34.3				64.0				63.0		45.1				52.0
Aminopenicillins							24.5										24.5
Amoxicillin			62.0							0.2						46.0	36.1
Amoxicillin/clavulanic acid			39.0						20.0		19.8						24.6
Ampicillin			83.4				20.8	97.7	84.0		26.4				92.1		67.4
Ampicillin/sulbactam	26.6		57.5								52.1						40.7
Aztreonam			22.1		20.4						36.0						26.2
Benzylpenicillin															1.0		1.0
Carbapenems	55.5		8.6	74.1	6.9						23.4		33.3	53.8			33.6
Cefaclor									55.5								55.5
Cefazolin			62.3								69.7						66.0
Cefepime	56.7		8.8		18.2						16.4		22.0				27.7
Cefoperazone/sulbactam	59.0																59.0
Cefotaxime	100.0		27.3									11.0	100.0			35.1	46.8
Cefotetan			0.6								20.0						10.3
Cefoxitin			17.0										100.0		25.7		47.6
Ceftazidime	66.4		25.3		24.8						29.5		25.3				32.3
Ceftazidime/cefepime	95.2												22.9				59.1
Ceftriaxone	62.8		41.3		20.8	25.4					37.6		91.4			38.2	45.6
Cefuroxime			7.0						32.5		19.5						19.7
Cephalosporins			87.4								68	0	0	48.5	0	0	76.8
Chloramphenicol									3.9							6.0	5.0
Ciprofloxacin	56.2	57.8	36.3		1.0	5.1		86.0			20.1	17.0	25.4	54.7	21.3		32.4
Clarithromycin										21.4							21.4
Clindamycin															25.8		25.8
Clindamycin		72.3													37.2		54.8
Colistin	3.8										30.1		7.0				14.1
Doripenem	24.4										33.4		8.1				24.8
Ertapenem				18	10						27.5		43.5			0.3	22.7
Erythromycin								88							27.8	55.8	51.1
ESBLs			18.2			26.7					34.3			25.0			26.8
Fluoroquinolones	71.7		39.5			16.0					42.7		38.8		81.5		48.3
Fosfomycin			1								27.9						14.5
Fosfomycin			14								80						47
Gentamicin	40.4	50.7	22.5		4.5		59.1				19.1		21.6	29.2	28.3		27.6
Glycopeptides								33.0									33.0
Imipenem	51.4		0.6		8.3						19.9		27.7				27.2
Imipenem/cilastatin													13				13
Levofloxacin	21.2		46.2		1.2			70.0		15.8	13.5		30.3		17.9	0.3	26.9
Linezolid	11.9	1															6.5
Meropenem	57.8		1.4			7.9					11.6		24.3			36.6	28.7
Methicillin															44.9		44.9
Metronidazole										38.9							38.9
Moxifloxacin															1.7		1.7
Multidrug	44.3		24.0								49.1		28.8				39.5
Nalidixic acid			52														52
Nitrofurantoin			4.6			3		5.9			10.4						5.4
Ofloxacin									0.3								0.3
Oxacillin		89.7													36.6		45.4
Penicillin			67					98.1							93	16.7	58.3
Piperacillin													9				9
Piperacillin/tazobactam	63.0		14.9		13.9	12.2					30.4		27.2	8.8			29.4
Rifampicin		13.6								0.9					17.85		13.61
Teicoplanin							28.0										28.0
Tetracycline								75.0	5.8						34.0	94.0	48.6
Tigecycline	6.1					0.8					6.9						5.6
Tobramycin	14.2		15.4								15.0		33.7				19.5
TXS	54	58.1	45.2		13.5	18.7					42.0				5.2	75.5	37.6
Vancomycin		6.5					23.3	33.2							10.4		21.3
Beta-lactam/beta-lactamase inhibitor			64.3				14.0										39.1
Total average (%)	52.6	43.7	27.4	46.1	12.8	17.7	33.4	59.4	28.9	15.4	28.8	14.0	29.0	48.6	29.7	36.7	38.4

ESBLs: extended-spectrum beta-lactam/beta lactamase-inhibitor; TXS: trimethoprim sulfamethoxazole; 3GC: third-generation cephalosporins.

**Table 3:** Distribution of correlation between ABC and antibiotic-resistant bacteria, 2016–2022 (*N* = 58).

ABC	Resistant antibiotic	Bacterial resistance to antibiotic in question (±)							
		*P. aeruginosa*	*K. pneumonia*	*E. coli*	*N. gonorrhoeae*	*A. baumannii*	*E. cloacae*	MRSA	Others (11)
Positive and negative correlation between ABC and rate of resistance		73/14	63/14	68/10	8	53/9	12(5)	15(11)	29

Overall correlation between ABC and rate of resistance		87	77	78	8	62	17	26	29

Carbapenem	Carbapenem	6(2)	8(1)	3		11	3	1	1
	Levofloxacin			0/1					0
	Imipenem	4(1)				2			0
	Ceftazidime	1	0/1						0
	Amikacin						1		0
	Fluoroquinolone	1	1						0
	3GC		1	1					0
	Meropenem	2							0
	Ciprofloxacin			0/1					0

Monobactam	Monobactam								
	Carbapenems					1	2		

Meropenem	Meropenem	3				1		1	0
	Piperacillin/tazobactam								1
	Carbapenems	2							0

Cefepime/Cefotaxime	Cefepime/Cefotaxime	1		2(1)		2			1

Imipenem	Imipenem	1(1)	1			1			0
	Meropenem		1						
	Piperacillin/tazobactam	1							
	Carbapenems	1							0

Imipenem/cilastatin	Imipenem/cilastatin	1	1	1				0/1	0

Piperacillin/tazobactam	Piperacillin/tazobactam	4		2		2	0/1	0/1	3
	Imipenem		1						
	Meropenem		1						
	Aminoglycoside		1						0

Linezolid	Linezolid								1

Fluroquinolone	Fluroquinolone	3	3(1)	5	1	1			3
	Trimethoprim/sulfamethoxazole		1						0
	Levofloxacin			3		2			1
	Imipenem	1				0/1			0
	Ceftazidime		2						0
	Amikacin						1		0
	Carbapenem		0/1	1(1)					1
	Ciprofloxacin	2(2)	3(1)	3(1)	1	3	0/1	1(2)	3
	3GC		1	2					0

Levofloxacin	Levofloxacin	2		1		1		1	0
	Fluoroquinolone	1	1	1					0
	Carbapenem		1						0

Ciprofloxacin	Ciprofloxacin	2(1)	2	3		1 (1)			1
	Carbapenem		0/1						0
	Meropenem					1			
	Imipenem					1			
	Piperacillin/tazobactam								
	Fluoroquinolone			1					0

Norfloxacin	Norfloxacin								0
	Carbapenem		0/1	1					0

Ofloxacin	Ofloxacin								0
	Carbapenem		0/1	0/1					0

Beta-lactam/beta-lactamase inhibitor	Beta-lactam/beta-lactamase inhibitor		1	2					0
	Carbapenem			1		4	2(1)		0
	Levofloxacin		2						0
	Imipenem	2				1			0
	Piperacillin/tazobactam	0/1							0
	Ceftazidime	4							0

Amoxicillin/clavulanate	Amoxicillin/clavulanate	1	1(1)	2				1(2)	2
	3GC		1						0

Cephalosporin	Cephalosporin	1	7(1)	7	1	1		2	0
	Amoxicillin/clavulanate			1					0
	Trimethoprim/sulfamethoxazole			1					0
	Levofloxacin		1			1			0
	3GC		1	2					0
	Ciprofloxacin				1				0
	Carbapenem		1			3			0
	Piperacillin/tazobactam	2	0/1						0
	Cefotaxime			2					
	Cefepime	0/1	2	2(1)					
	Ceftazidime		0/1						0

Aminoglycosides	Aminoglycosides	1				2(2)		0/1	0
	3GC		1(1)	1(1)					0
	Ciprofloxacin			1(1)	1				0
	Imipenem	1				1			0
	Ceftazidime		1						0
	Amikacin	2					1		0
	Gentamicin	2							0
	Carbapenems	1							0

Macrolides	Macrolides				1				2
	Cefixime				1				0
	Ciprofloxacin				1				0
	Clarithromycin								2

Tigecycline	Tigecycline					1			0
	Piperacillin/tazobactam	0/1							0

Amikacin	Amikacin	3(2)				0/1			0
	Aminoglycosides	1							0
	Imipenem	1 (1)				0/1			0
	Meropenem					0/1			0

Gentamicin	Gentamicin	1	1(1)	1		1			1
	Aminoglycosides	1							0

Fosfomycin	Fosfomycin			1		1			0

Oxacephems	Oxacephems					1			0
	Levofloxacin			1					0
	Imipenem	2				0/1			0
	Ceftazidime		1						0

Sulfonamides	Sulfonamides		2	2				1	0
	Imipenem	1							0
	Ceftazidime		1						0

All antibiotics	All antibiotics		1	1			1		2
	Fluoroquinolone		1	1		1			0
	Levofloxacin			1					0
	3GC		1	1					0
	Carbapenem		1	1					0
	Cloxacillin							1	0
	Macrolides								1

*β*-Lactam antibiotics	*β*-Lactam antibiotics		1						0
	3GC		2	1					0
	Carbapenem	1	1						0

Cloxacillin	Cloxacillin							1	0

Tetracycline	Tetracycline								0
	Fluoroquinolone			1					0
	Nalidixic acid			1					0
	Carbapenem					1			0

Ceftazidime	Ceftazidime	1							
	Imipenem					1			
	Meropenem	1				1			

Glycopeptides	Glycopeptides	1					1	0/2	1
	Amikacin			0/1		0/1	0/1		0
	Gentamicin						0/1	1(1)	3
	Carbapenem		1			1			
	Piperacillin/tazobactam	1						1	0
	Extended-spectrum cephalosporin			1					
	Amikacin	1		1		1			1
	Oxacillin							1	0

Others				1				2 (1)	0

3GC: third-generation cephalosporins.

## Data Availability

Since all data are included in the review, no additional data is required to support the findings of this study.

## Supplementary Materials

Supplementary Materials Annex 1: summary of relationship between antibiotic consumption and antibiotic resistance, 2016-2022, (N=58).

## References

[1] WHO (2019). *Antibiotic Stewardship Programs in Health-Care Facilities in Low- and Middle-Income Countries. A Practical Toolkit*.

[2] Obe D. N. (2018). *Antibiotic Stewardship from Principles to Practice*.

[3] Dhingra S., Rahman N. A. A., Peile E. (2020). Microbial resistance movements: an overview of global public health threats posed by antimicrobial resistance, and how best to counter. *Frontiers in Public Health*.

[4] WHO (2020). Antibiotic resistance. https://www.who.int/news-room/fact-sheets/detail/antibiotic-resistance.

[5] Spera A. M., Esposito S., Pagliano P. (2019). Emerging antibiotic resistance: carbapenemase-producing Enterobacteria. New bad bugs, still no new drugs. *Infections in Medicine*.

[6] CDC (2019). *Antibiotic Resistance Threats in the United States, 2019*.

[7] World Bank (2016). *Drug-Resistant Infections: A Threat to Our Economic Future (Discussion Draft)*.

[8] Thabit A. K., Shea K. M., Guzman O. E., Garey K. W. (2021). Antibiotic utilization within 18 community hospitals in the United States: a 5-year analysis. *Pharmacoepidemiology and Drug Safety*.

[9] Dellit T. H., Owens R. C., McGowan J. E. (2007). Infectious diseases society of America and the society for healthcare epidemiology of America guidelines for developing an institutional program to enhance antimicrobial stewardship. *Clinical Infectious Diseases*.

[10] Sedláková M. H., Urbánek K., Vojtová V., Suchánková H., Imwensi P., Kolář M. (2014). Antibiotic consumption and its influence on the resistance in Enterobacteriaceae. *BMC Research Notes*.

[11] EFMHACA (2018). A practical guide to antibiotic stewardship program in Ethiopian hospitals.

[12] Lee H.-S., Loh Y.-X., Lee J.-J., Liu C.-S., Chu C. (2015). Antibiotic consumption and resistance in five Gram-negative bacterial species in a hospital from 2003 to 2011. *Journal of Microbiology, Immunology, and Infection*.

[13] McLaughlin M., Advincula M. R., Malczynski M., Qi C., Bolon M., Scheetz M. H. (2013). Correlations of antibiotic use and carbapenem resistance in *Enterobacteriaceae*. *Antimicrobial Agents and Chemotherapy*.

[14] Wu H.-H., Liu H.-Y., Lin Y.-C., Hsueh P.-R., Lee Y.-J. (2016). Correlation between levofloxacin consumption and the incidence of nosocomial infections due to fluoroquinolone-resistant *Escherichia coli*. *Journal of Microbiology, Immunology, and Infection*.

[15] Cao J., Song W., Gu B. (2013). Correlation between carbapenem consumption and antibiotic resistance rates of *Acinetobacter baumannii* in a university-affiliated hospital in China. *The Journal of Clinical Pharmacology*.

[16] Weng T.-C., Chen Y.-H., Lee C.-C. (2011). Correlation between fluoroquinolone consumption in hospitals and ciprofloxacin resistance amongst *Pseudomonas aeruginosa* isolates causing healthcare-associated infections, Taiwan, 2000–2009. *International Journal of Antimicrobial Agents*.

[17] Lai C. C., Wang C. Y., Chu C. C. (2011). Correlation between antimicrobial consumption and resistance among *Staphylococcus aureus* and enterococci causing healthcare-associated infections at a university hospital in Taiwan from 2000 to 2009. *European Journal of Clinical Microbiology and Infectious Diseases*.

[18] Versporten A., Zarb P., Caniaux I. (2018). Antimicrobial consumption and resistance in adult hospital inpatients in 53 countries: results of an internet-based global point prevalence survey. *Lancet Global Health*.

[19] Schober P., Boer C., Schwarte L. A. (2018). Correlation coefficients: appropriate use and interpretation. *Anesthesia and Analgesia*.

[20] Hirayama S., Yasui K., Murakami H. (2018). A new carbapenem drug dosage metric for carbapenem usage and correlation with carbapenem resistance of *Pseudomonas aeruginosa*. *Journal of Infection and Chemotherapy*.

[21] Zhang D., Hu S., Sun J. (2019). Antibiotic consumption versus the prevalence of carbapenem-resistant Gram-negative bacteria at a tertiary hospital in China from 2011 to 2017. *Journal of Infection and Public Health*.

[22] Barberi G., De Cola M. C., Dell’Utri D. C. (2017). Antimicrobial consumption and antimicrobial resistance: a snapshot of an Italian neuromuscular rehabilitation center. *New Microbiologica*.

[23] Kenyon C., Buyze J., Wi T. (2018). Antimicrobial consumption and susceptibility of Neisseria gonorrhoeae: a global ecological analysis. *Frontiers of Medicine*.

[24] Rosado-Rosado D. A., Arias-Flores R., Vázquez-Rosales J. G., Robles-Ramírez R. J., Del Campo-Ortega R., Ascencio-Montiel I. J. (2021). Antimicrobial resistance and antibiotic consumption in a third level pediatric hospital in Mexico City. *The Journal of Infection in Developing Countries*.

[25] Guo W., Sun F., Liu F., Cao L., Yang J., Chen Y. (2019). Antibiotic resistance surveillance and prediction of Gram-negative bacteria based on antibiotic consumption in a hospital setting: a 15-year retrospective study. *Medicine*.

[26] Yang P., Chen Y., Jiang S., Shen P., Lu X., Xiao Y. (2018). Association between antibiotic consumption and the rate of carbapenem-resistant Gram-negative bacteria from China based on 153 tertiary hospitals data in 2014. *Antimicrobial Resistance and Infection Control*.

[27] Yang P., Chen Y., Jiang S., Shen P., Lu X., Xiao Y. (2020). Association between the rate of fluoroquinolones-resistant gram-negative bacteria and antibiotic consumption from China based on 145 tertiary hospitals data in 2014. *BMC Infectious Diseases*.

[28] Yang P., Chen Y., Jiang S., Shen P., Lu X., Xiao Y. (2020). Association between the rate of third generation cephalosporin-resistant *Escherichia coli* and *Klebsiella pneumoniae* and antibiotic consumption based on 143 Chinese tertiary hospitals data in 2014. *European Journal of Clinical Microbiology and Infectious Diseases*.

[29] Barchitta M., Maugeri A., La Rosa M. C. (2021). Carbapenem Consumption and Rate of carbapenem resistant gram-negative bacteria: results from the Sicilian Surveillance System. *Annals of Hygiene, Preventive and Community Medicine*.

[30] Terahara F., Nishiura H. (2019). Carbapenem-resistant *Pseudomonas aeruginosa* and carbapenem use in Japan: an ecological study. *Journal of International Medical Research*.

[31] Seo M. R., Kim B., Kim J., Pai H. (2019). Change in antimicrobial susceptibility and PCR ribotypes of Clostridioides difficile in a hospital over 5 years: correlation analysis with antimicrobial consumption. *International Journal of Antimicrobial Agents*.

[32] Kousovista R., Athanasiou C., Liaskonis K., Ivopoulou O., Ismailos G., Karalis V. (2021). Correlation between *Acinetobacter baumannii* resistance and hospital use of meropenem, cefepime, and ciprofloxacin: time series analysis and dynamic regression models. *Pathogens*.

[33] Mascarello M., Simonetti O., Knezevich A. (2017). Correlation between antibiotic consumption and resistance of bloodstream bacteria in a University Hospital in North Eastern Italy, 2008–2014. *Infection*.

[34] Mladenovic-Antic S., Kocic B., Velickovic-Radovanovic R. (2016). Correlation between antimicrobial consumption and antimicrobial resistance of *Pseudomonas aeruginosa* in a hospital setting: a 10-year study. *Journal of Clinical Pharmacy and Therapeutics*.

[35] Prakobsrikul N., Malathum K., Santanirand P., Chumnumwat S., Piebpien P., Montakantikul P. (2019). Correlation between antibiotic consumption and the prevalence of carbapenem‐resistant *Escherichia coli* and carbapenem-resistant *Klebsiella pneumoniae* at a university hospital in Thailand. *Journal of Clinical Pharmacy and Therapeutics*.

[36] Zivanovic V., Gojkovic-Bukarica L., Scepanovic R. (2017). Differences in antimicrobial consumption, prescribing and isolation rate of multidrug resistant *Klebsiella pneumoniae*, *Pseudomonas aeruginosa* and Acinetobacter baumannii on surgical and medical wards. *PLoS One*.

[37] Lai C.-C., Shi Z.-Y., Chen Y.-H., Wang F.-D. (2016). Effects of various antibiotic stewardship programs on antibiotic usage and resistance among common gram-negative bacilli causing health care-associated infections: a multicenter comparison. *Journal of Microbiology, Immunology, and Infection*.

[38] Olearo F., Both A., Belmar Campos C. (2021). Emergence of linezolid-resistance in vancomycin-resistant *Enterococcus faecium* ST117 associated with increased linezolid-consumption. *International Journal of Medical Microbiology*.

[39] Popović R., Tomić Z., Tomas A. (2020). Five-year surveillance and correlation of antibiotic consumption and resistance of Gram-negative bacteria at an intensive care unit in Serbia. *Journal of Chemotherapy*.

[40] Terahara F., Nishiura H. (2019). Fluoroquinolone consumption and *Escherichia coli* resistance in Japan: an ecological study. *BMC Public Health*.

[41] Shakoor S., Tahseen S., Jabeen K. (2016). Fluoroquinolone consumption and-resistance trends in *Mycobacterium tuberculosis* and other respiratory pathogens: ecological antibiotic pressure and consequence s in Pakistan, 2009–2015. *International Journal of Mycobacteriology*.

[42] Wang H., Wang H., Yu X. (2019). Impact of antimicrobial stewardship managed by clinical pharmacists on antibiotic use and drug resistance in a Chinese hospital, 2010–2016: a retrospective observational study. *BMJ Open*.

[43] Ruiz J., Gordon M., Villarreal E. (2019). Influence of antibiotic pressure on multi-drug resistant *Klebsiella pneumoniae* colonisation in critically ill patients. *Antimicrobial Resistance and Infection Control*.

[44] Baditoiu L., Axente C., Lungeanu D. (2017). Intensive care antibiotic consumption and resistance patterns: a cross-correlation analysis. *Annals of Clinical Microbiology and Antimicrobials*.

[45] Cusini A., Herren D., Bütikofer L., Plüss-Suard C., Kronenberg A., Marschall J. (2018). Intra-hospital differences in antibiotic use correlate with antimicrobial resistance rate in *Escherichia coli* and *Klebsiella pneumoniae*: a retrospective observational study. *Antimicrobial Resistance and Infection Control*.

[46] Marquet A., Vibet M. A., Caillon J. (2018). Is there an association between use of amoxicillin-clavulanate and resistance to third-generation cephalosporins in *Klebsiella pneumoniae* and *Escherichia coli* at the hospital level?. *Microbial Drug Resistance*.

[47] Kastrin T., Paragi M., Erculj V., Žohar Čretnik T., Bajec T., Cižman M. (2019). Lack of correlation between reduced outpatient consumption of macrolides and macrolide resistance of invasive Streptococcuspneumoniae isolates in Slovenia during 1997–2017. *Journal of Global Antimicrobial Resistance*.

[48] McDonnell L., Armstrong D., Ashworth M., Dregan A., Malik U., White P. (2017). National disparities in the relationship between antimicrobial resistance and antimicrobial consumption in Europe: an observational study in 29 countries. *Journal of Antimicrobial Chemotherapy*.

[49] Kenyon C., Buyze J., Spiteri G., Cole M. J., Unemo M. (2020). Population level consumption of cephalosporins and macrolides may select for reduced antibiotic susceptibility to unrelated antibiotics in *Neisseria gonorrhoeae*: an ecological analysis. *The Journal of Infectious Diseases*.

[50] Kenyon C. (2019). Population-level macrolide consumption is associated with clarithromycin resistance in *Helicobacter pylori*: an ecological analysis. *International Journal of Infectious Diseases*.

[51] Kenyon C. (2019). Prevalence of macrolide resistance in *Treponema pallidum* is associated with macrolide consumption. *Journal of Medical Microbiology*.

[52] Djordjevic Z. M., Folic M. M., Jankovic S. M. (2017). Previous antibiotic exposure and antibiotic resistance patterns of *acinetobacter* spp. and *Pseudomonas aeruginosa* isolated from patients with nosocomial infections. *Balkan Medical Journal*.

[53] Ribeiro Á. C. D. S., Crozatti M. T. L., Silva A. A., Macedo R. S., Machado A. M. D. O., Silva A. T. D. A. P. (2019). *Pseudomonas aeruginosa* in the ICU: prevalence, resistance profile, and antimicrobial consumption. *Journal of the Brazilian Society of Tropical Medicine*.

[54] Ryu S., Klein E. Y., Chun B. C. (2018). Temporal association between antibiotic use and resistance in *Klebsiella pneumoniae* at a tertiary care hospital. *Antimicrobial Resistance and Infection Control*.

[55] Nadia J., Wejdene M., Remy A B. (2019). Temporal variation in antibiotic resistance of *Acinetobacter baumannii* in a teaching hospital in Tunisia: correlation with antibiotic consumption. *The Open Microbiology Journal*.

[56] Batard E., Vibet M.-A., Thibaut S. (2018). Tetracycline use in the community may promote decreased susceptibility to quinolones in *Escherichia coli* isolates. *European Journal of Clinical Microbiology and Infectious Diseases*.

[57] Hongchumpae O., Santimaleeworagun W. (2016). The correlation between defined daily dose/1000 patient-day of antibiotics 3 the resistance rate of *P. aeruginosa* and A. Baumannii: a case study at hua-hin hospital. *Thai Pharmaceutical and Health Science Journal*.

[58] Asencio Egea M. A., Huertas Vaquero M., Carranza González R., Herráez Carrera Ó., Redondo González O., Arias Arias Á. (2018). Trend and seasonality of community-acquired *Escherichia coli* antimicrobial resistance and its dynamic relationship with antimicrobial use assessed by ARIMA models. *Infectious Diseases and Clinical Microbiology*.

[59] Granov D., Ljubovic A. D., Zec S. L., Granov N., Hukic M. (2016). The impact of antibiotic consumption on development of *acinetobacter baumannii resistance*. *Materia Socio Medica*.

[60] Liang C., Zhang X., Zhou L., Meng G., Zhong L., Peng P. (2021). Trends and correlation between antibacterial consumption and carbapenem resistance in gram-negative bacteria in a tertiary hospital in China from 2012 to 2019. *BMC Infectious Diseases*.

[61] Kim B., Kim Y., Hwang H. (2018). Trends and correlation between antibiotic usage and resistance pattern among hospitalized patients at university hospitals in Korea, 2004 to 2012. A nationwide multicenter study. *Medicine*.

[62] Wang R., Yang Q., Zhang S., Hong Y., Zhang M., Jiang S. (2019). Trends and correlation of antibiotic susceptibility and antibiotic consumption at a large teaching hospital in China (2007–2016): a surveillance study. *Therapeutics and Clinical Risk Management*.

[63] Wushouer H., Zhang Z.-X., Wang J.-H. (2018). Trends and relationship between antimicrobial resistance and antibiotic use in Xinjiang Uyghur Autonomous Region, China: based on a 3 year surveillance data, 2014–2016. *Journal of Infection and Public Health*.

[64] Zhang D., Cui K., Wang T. (2019). Trends in and correlations between antibiotic consumption and resistance of *Staphylococcus aureus* at a tertiary hospital in China before and after introduction of an antibiotic stewardship program. *Epidemiology and Infection*.

[65] Kim Y. A., Park Y. S., Youk T., Lee H., Lee K. (2018). Trends in South Korean antibiotic use and association with changes in *Escherichia coli* resistance rates: 12-year ecological study using a nationwide surveillance and antibiotic prescription database. *PLoS One*.

[66] Martinez E. P., van Rosmalen J., Bustillos R., Natsch S., Mouton J. W., Verbon A. (2020). Trends, seasonality and the association between outpatient antibiotic use and antimicrobial resistance among urinary bacteria in The Netherlands. *Journal of Antimicrobial Chemotherapy*.

[67] Wang A., Daneman N., Tan C., Brownstein J. S., MacFadden D. R. (2017). Evaluating the relationship between hospital antibiotic use and antibiotic resistance in common nosocomial pathogens. *Infection Control and Hospital Epidemiology*.

[68] Chen S., Li Z., Shi J. (2022). A nonlinear time-series analysis to identify the thresholds in relationships between antimicrobial consumption and resistance in a Chinese tertiary hospital. *Infectious Disease and Therapy*.

[69] Pérez-Lazo G., Abarca-Salazar S., Lovón R. (2021). Antibiotic consumption and its relationship with bacterial resistance profiles in ESKAPE pathogens in a Peruvian hospital. *Antibiotics*.

[70] Barnsteiner S., Baty F., Albrich W. C. (2021). Antimicrobial resistance and antibiotic consumption in intensive care units, Switzerland, 2009 to 2018. *Euro Surveillance*.

[71] Cižman M., Mioč V., Bajec T., Paragi M., Kastrin T., Gonçalves J. (2021). Correlation between antibiotic consumption and resistance of invasive *Streptococcus pneumoniae*. *Antibiotics*.

[72] Huang H.-W., Liu H.-Y., Chuang H.-C. (2023). Correlation between antibiotic consumption and resistance of *Pseudomonas aeruginosa* in a teaching hospital implementing an antibiotic stewardship program: a longitudinal observational study. *Journal of Microbiology, Immunology, and Infection*.

[73] Khare S., Diwan V., Pathak A., Purohit M. R., Stålsby Lundborg C. (2022). Correlation between individual child-level antibiotic consumption and antibiotic-resistant among commensal *Escherichia coli*: results from a cohort of children aged 1–3 Years in rural ujjain India. *Infection and Drug Resistance*.

[74] Manoharan-Basil S., Gonzalez N., Kenyon C. (2022). Country-level association between antibiotic consumption and resistance in Neisseria meningitidis: an ecological study. *Journal of Infection and Public Health*.

[75] Megraud F., Bruyndonckx R., Coenen S. (2021). *Helicobacter pylori* resistance to antibiotics in Europe in 2018 and its relationship to antibiotic consumption in the community. *Gut*.

[76] Hayajneh W. A., Al-Azzam S., Yusef D. (2021). Identification of thresholds in relationships between specific antibiotic use and carbapenem-resistant Acinetobacter baumannii (CRAb) incidence rates in hospitalized patients in Jordan. *Journal of Antimicrobial Chemotherapy*.

[77] Gong W., Tang W., Luo L. (2022). Trends and correlation between antimicrobial resistance and antibiotics consumption in a specialist children’s hospital from 2016 to 2021. *Infection and Drug Resistance*.

[78] Asokan G. V., Ramadhan T., Ahmed E., Sanad H. (2019). WHO global priority pathogens list: a bibliometric analysis of medline PubMed for knowledge mobilization to infection prevention and control practices in Bahrain. *Oman Medical Journal*.

[79] Balasubramanian R., Van Boeckel T. P., Carmeli Y., Cosgrove S., Laxminarayan R. (2023). Global incidence in hospital-associated infections resistant to antibiotics: an analysis of point prevalence surveys from 99 countries. *PLoS Medicine*.

[80] Pandey R., Mishra S. K., Shrestha A. (2021). Characterisation of ESKAPE pathogens with special reference to multidrug resistance and biofilm production in a Nepalese hospital. *Infection and Drug Resistance*.

[81] Cai Y., Hoo G. S. R., Lee W. (2022). Estimating the economic cost of carbapenem resistant Enterobacterales healthcare associated infections in Singapore acute-care hospitals. *PLOS Global Public Health*.

[82] Global (2023). Global point prevalence survey: impact and Value. https://www.global-pps.com/wp-content/uploads/2023/03/2023-GLOBAL-PPS-Selection-of-Publications-FINAL-INTERACTIVE-LINKS-V2-1.pdf.

[83] O’Neill J. (2016). *Review on Abr: Antibiotic Resistance: Tackling A Crisis for the Health and Wealth of Nations*.

[84] Van Boeckel T. P., Gandra S., Ashok A. (2014). Global antibiotic consumption 2000 to 2010: an analysis of national pharmaceutical sales data. *The Lancet Infectious Diseases*.

[85] Browne A. J., Chipeta M. G., Haines-Woodhouse G. (2021). Global antibiotic consumption and usage in humans, 2000–18: a spatial modelling study. *The Lancet Planetary Health*.

[86] Tan S. Y., Khan R. A., Khalid K. E., Chong C. W., Bakhtiar A. (2022). Correlation between antibiotic consumption and the occurrence of multidrug-resistant organisms in a Malaysian tertiary hospital: a 3-year observational study. *Scientific Reports*.

[87] Gebreyohannes G., Nyerere A., Bii C., Sbhatu D. B. (2019). Challenges of intervention, treatment, and antibiotic resistance of biofilm-forming microorganisms. *Heliyon*.

[88] Sabrot A. V., Mohamed A. A., Faradjeva E. (2021). Mechanisms and impact of biofilms and targeting of biofilms using bioactive compounds—a review. *Medicine*.

[89] Bell B. G., Schellevis F., Stobberingh E., Goossens H., Pringle M. (2014). A systematic review and meta-analysis of the effects of antibiotic consumption on antibiotic resistance. *BMC Infectious Diseases*.

[90] Wu C.-T., Chen C.-L., Lee H.-Y. (2017). Decreased antibiotic resistance and defined daily doses after implementation of a clinical culture-guided antibiotic stewardship program in a local hospital. *Journal of Microbiology, Immunology, and Infection*.

[91] Cunha B. (2002). Strategies to control antibiotic resistance. *Seminars in Respiratory Infections*.

[92] Tamma P. D., Avdic E., Keenan J. F. (2017). What is the more effective antibiotic stewardship Intervention: pre-prescription Authorization or Post-prescription review with Feedback?. *Clinical Infectious Diseases: An Official Publication of the Infectious Diseases Society of America*.

