# Supplementary material for: Relationship between Antibiotic Consumption and Resistance: A Systematic Review
Source: Can J Infect Dis Med Microbiol. 2024 Mar 5;2024:9958678. doi: 10.1155/2024/9958678 (PMC10932619; doi:10.1155/2024/9958678)
Supplement: Supplementary Materials — Annex 1: summary of relationship between antibiotic consumption and antibiotic resistance, 2016-2022, (N=58). [file 9958678.f1.docx]

Annex 1: Summary of relationship between antibiotic consumption and antibiotic resistance, 2016-2022, (N=58)

| **Study** | **Country, year** | **Study design** | **Setting** | **Antibiotic consumption** | **Resistant microorganism** | **Correlation coefficient** | **P.value** | **Author (reference** |
| --- | --- | --- | --- | --- | --- | --- | --- | --- |
| A new carbapenem drug dosage metric for carbapenem usage and correlation with carbapenem resistance of P. aeruginosa | Japan, 2018 | Trend analysis | University Hospital | carbapenem | P. aeruginosa | R=-0.915 | P<0.001 | Hirayama S, et al (20) |
|  |  |  |  |  |  |  |  |  |
| Antibiotic consumption versus the prevalence of carbapenem-resistant Gram-negative bacteria at a tertiary hospital in China from 2011 to 2017 | China, 2019 | Trend analysis | University Hospital | Carbapenem | K. pneumonia | R= 0.543 | P=0.003 | Di Zhang D., et al (21). |
| Antimicrobial consumption and antimicrobial resistance: a snapshot of an Italian neuromuscular rehabilitation center | Italy, 2017 | Retrospective, consecutive | Rehabilitation center | B-Lactam/Inhibitor | *methicilin resistant S. aureus* | 0.996 | 0.03 | Barberi G., et al (22) |
|  |  |  |  | B-Lactam/Inhibitor | aminoglycoside resistant K. pneumoniae | -0.991 | 0.04 |  |
|  |  |  |  | B-Lactam/Inhibitor | Fluoroquinolones resistant K. pneumoniae | -0.997 | 0.002 |  |
|  |  |  |  | B-Lactam/Inhibitor | *Carbapenems resistant E. coli* | 0.993 | 0.03 |  |
|  |  |  |  | B-Lactam/Inhibitor | Piperacillin/tazobactam resistant E. coli | -0.993 | 0.03 |  |
|  |  |  |  | Piperacillin /Tazobactam | Aminoglycosides esistant K. pneumoniae | 0.993 | 0.03 |  |
|  |  |  |  | Piperacillin /Tazobactam | 3GC resistant P. mirabilis | -0.993 | 0.03 |  |
|  |  |  |  | Piperacillin / Tazobactam | 4GC resistant P. mirabilis | -0.999 | 0.01 |  |
|  |  |  |  | 3GC | Carbapenems resistant A. baumannii | -1 | 0.009 |  |
|  |  |  |  | 3GC | Amoxicillin/clavulanate resistant E. coli | 1 | 0.001 |  |
|  |  |  |  | 3GC | *Hlr-gentamcin resistanr E. faecalis* | -1 | 0.005 |  |
|  |  |  |  | Carbapenems | 3GC resistant P. aeruginosa | 0.995 | 0.03 |  |
|  |  |  |  | Carbapenems | Aminoglycosides resistant P. aeruginosa | 0.99 | 0.04 |  |
|  |  |  |  | Fluoroquinolones | Trimethoprim/sulfamethoxazole resistant *K. pneumoniae* | 0.992 | 0.003 |  |
|  |  |  |  | Aminoglycosides | 3GC resistant E coli | 0.997 | 0.002 |  |
|  |  |  |  | Aminoglycosides | Fluoroquinolones resistant E coli | 0.997 | 0.002 |  |
|  |  |  |  | Colistin | Amoxicillin/clavulanateresistant E coli | -0.999 | 0.001 |  |
|  |  |  |  | Colistin | Trimethoprim/sulfamethoxazole resistant *E coli* | 0.991 | 0.04 |  |
| Antimicrobial Consumption and Susceptibility of Neisseria gonorrhoeae: A Global Ecological | Global, 2018 | Retrospective | population level consumption of antimicrobials | Cephalosporin | *N. gonorrhoeae* | 0.9 | 0.0003 | *Kenyon C., et al (23)* |
|  |  |  |  | Azithromycin |  | 0.6 | 0.0005 |  |
|  |  |  |  | Ciprofloxacin |  | 0.26 | 0.02 |  |
| Antimicrobial resistance and antibiotic consumption in a third level pediatric hospital in Mexico City | Mexico, 2021 | Retrospective | Pediatric | Amikacin | *P. aeruginosa* | r = 0.95, | p = 0.05 | Rosado-Rosado *et al (24)* |
|  |  |  | Hospital |  |  |  |  |  |
| Antimicrobial resistance surveillance and prediction of Gram-negative bacteria based on antimicrobial consumption in a hospital setting A 15-year retrospective study | China, 2019 | Retrospective | University Hospital | carbapenem | Imipenem resistance A. baumannii | r=0.954, | P=P0001 | Goe W., et al (25)* |
|  |  |  |  | aminoglycosides |  | r=-0.811, | P=.001 |  |
|  |  |  |  | quinolone |  | r=-0.864, | P=0.0001 |  |
|  |  |  |  | Oxacephems |  | R=-0.650 | P=.016 |  |
|  |  |  |  | Consumption | Levofloxacin resistance E. coli | r=0.620, | *P*=.024 |  |
|  |  |  |  | combinations |  |  |  |  |
|  |  |  |  | oxacephems |  | r=0.724, | *P*=.005 |  |
|  |  |  |  | Quinolones |  | R=0.732 | P= 0.004 |  |
|  |  |  |  | carbapenem |  | R=-0.659 | P=0.014 |  |
|  |  |  |  | Oxacephems |  | R=0.724 | P=0 .005 |  |
|  |  |  |  | b-lactamase inhibitor combinations | Imipenem resistance P. aeruginosa | r=0.715 | P=.006 |  |
|  |  |  |  | Consumption of oxacephems |  | r=0.720, | P=.006 |  |
|  |  |  |  | Consumption of sulfonamides |  | r=0.691, | P=.009 |  |
|  |  |  |  | Consumption of aminoglycosides |  | 0.916 | 0 |  |
|  |  |  |  | Consumption of Quinolones |  | 0.818 | 0.001 |  |
|  |  |  |  | Consumption of carbapenem |  | -0.86 | 0 |  |
|  |  |  |  | Aminoglycosides | Ceftazidime resistance K. pneumoniae | 0.609 | 0.027 |  |
|  |  |  |  | Quinolone |  | 0.844 | 0 |  |
|  |  |  |  | Carbapenem |  | -0.784 | 0 .002 |  |
|  |  |  |  | Oxacephems |  | 0.61 | 0.027 |  |
|  |  |  |  | Sulfonamides |  | 0.939 | 0.023 |  |
|  |  |  |  | Aminoglycosides | Amikacin resistant E. cloacae | 0.772 | 0.002 |  |
|  |  |  |  | Quinolone |  | 0.795 | 0.001 |  |
|  |  |  |  | Carbapenem | Imipenem resistance  K. pneumoniae | 0.921 | 0..000 |  |
|  |  |  |  | Carbapenem |  | 0.572 | 0.041 |  |
|  |  |  |  |  |  |  |  |  |
| Association between antibiotic consumption and the rate of carbapenem-resistant Gram-negative bacteria from China based on 153 tertiary hospitals data in 2014 | China, 2018 | Retrospecive | Tertiary hospitals | Carbapenem | Carbapenem resistant E. coli | R=0.271, | p < 0.01 | Yang P., et al (26) |
|  |  |  |  | Carbapenem | Carbapenem resistant K. pneumoniae | r = 0.427, | p < 0.01 |  |
|  |  |  |  | Carbapenem | Carbapenem resistant P. aeruginosa | r = 0.463, | p <0.01 |  |
|  |  |  |  | Carbapenem | Carbapenem resistant A. baumannii | r = 0.331 | p < 0.01 |  |
| Association between the rate of fluoroquinolones-resistant gram-negative bacteria and antibiotic consumption from China based on 145 tertiary hospitals data in 2014 | China, 2020 | Retrospecive | Tertiary hospitals | Fluroquinolones | Fluroquinolones Resistant of E. coli | r = 0.308, | p<0.01) | Yang P., et al (27) |
|  |  |  |  | Levofloxacin |  | r = 0.252 | p<0.01 |  |
|  |  |  |  | all antibiotics | Levofloxacin resistant A. baumannii | r = 0.282 | p<0.01 |  |
|  |  |  |  | Third-generation |  | r = 0.246, | p<0.01), |  |
|  |  |  |  | levofloxacin |  | r = 0.336, | p<0.01 |  |
|  |  |  |  | Fluroquinolones |  | r= 0.254 | P<0.01 |  |
|  |  |  |  | all antibiotics | fluoroquinolone resistant K. pneumoniae | 0.247, | p<0.01 |  |
|  |  |  |  | Carbapenems |  | r = 0.242, | p<0.01 |  |
|  |  |  |  | Fluroquinolones |  | r= 0.291, | p<0.01 |  |
|  |  |  |  | Levofloxacin |  | r = 0.260, | p<0.01 |  |
|  |  |  |  | all antibiotics | fluoroquinolone resistant P. aeruginosa | r = 0.260, | p<0.01 |  |
|  |  |  |  | Fluroquinolones |  | r = 0.319, | p<0.01 |  |
|  |  |  |  | Levofloxacin |  | r = 0.377, | p<0.01 |  |
| Association between the rate of third generation cephalosporin-resistant Escherichia coli and Klebsiella pneumoniae and antibiotic consumption based on 143 Chinese tertiary hospitals data in 2014 | China, 2020 | Retrospective | Tertiary hospitals | All antibiotics | 3GC-resistant E. coil | r = 0.252, | p < 0.01 | Yang P., et al (28) |
|  |  |  |  | β-Lactams including β-lactamase inhibitors | 3GC-resistant E. coil | r = 0.313, | p < 0.01 |  |
|  |  |  |  | β-Lactams alone | 3GC-resistant E. coil | r = 0.365, | p < 0.01 |  |
|  |  |  |  | Cephalosporins including β-lactamase inhibitors | 3GC-resistant E. coil | r = 0.398, | p < 0.01 |  |
|  |  |  |  | Cephalosporins | 3GC-resistant E. coil | r =0.374, | p < 0.01 |  |
|  |  |  |  | 3GC including β-lactamase inhibitors | 3GC-resistant E. coil | r = 0.321, | p < 0.01 |  |
|  |  |  |  | 3GC | 3GC-resistant E. coil | r = 0.343, | p < 0.01 |  |
|  |  |  |  | All antibiotics | 3GC-resistant K. pneumoniae | r = 0.200, | p < 0.05 |  |
|  |  |  |  | β-Lactams including β-lactamase inhibitors |  | r = 0.232, | p < 0.01 |  |
|  |  |  |  | 3GC including β-lactamase inhibitors |  | r =0.215, | p < 0.05 |  |
|  |  |  |  | 3GC |  | r = 0.383, | p < 0.01 |  |
|  |  |  |  | β-lactam-β-lactamase inhibitor combinations |  | r = 0.218, | p < 0.05 |  |
| Carbapenem Consumption and Rate of carbapenem resistant gram-negative bacteria: results from the Sicilian Surveillance System | Sicily, 2021 | *Retrospectively* | *Regional surveillance system for in hospital settings* | Beta-lactam | *Carbapenem resistant K.pneumonia* | R=0.529 | p<0.01 | Barchitta M., et al (29) |
|  |  |  |  | Carbapenem |  |  |  |  |
|  |  |  |  | all antibiotics | *Carbapenem resistant E. coli* | r=0.402 | p<0.05 |  |
| Carbapenem-resistant Pseudomonas aeruginosa and carbapenem use in Japan: an ecological study | Japan, 2019 | *Retrospective ecological study* | Health Insurance Claims | Carbapenem | Imipenem resistant P. aeruginosa | r=0.53 | P<0.01 | Terahara F., Nishiura H (30) |
| Change of antimicrobial susceptibility and PCR ribotypes of *Clostridioides difficile in a hospital over 5 years: correlation analyses with antimicrobial consumption consumption* | South Korea, 2019 | *Retrospective* | *University Hospital* | piperacillin/tazobactam | *C. difficile* | r=0.900, | *p*=0.037 | Seo M-R., et al (31) |
|  |  |  |  | Vancomycin | Resistant *C. difficile* | r=-1.000 | p <0.001 |  |
| Correlation between *Acinetobacter baumannii* Resistance and Hospital Use of Meropenem, Cefepime, and Ciprofloxacin:Time Series Analysis and Dynamic Regression Models | Greece, 2021 | *Retrospective,* time series models | Tertiary care hospital | meropenem | Rate of meropenem-resistant *A. baumannii* | 0.626 | *p* = 0.024 | **Kousovista R., et al (32)** |
|  |  |  |  | cefepime | cefepime-resistant *A. baumannii* | 0.66 | *p* = 0.028 |  |
|  |  |  |  | Ciprofloxacin | ciprofloxacin -resistant *A. baumannii* | 0.617 | *p* < 0.001 |  |
| Correlation between antibiotic consumption and resistance of bloodstream bacteria in a University Hospital in North Eastern Italy, 2008–2014 | Italy, 2017 | *Retrospective* | University hospital | penicillins | oxacillin resistance in S. aureus | r=-**0.9** | *p* = 0.002 | **Mascarello M., et al (33)** |
|  |  |  |  | amoxicillin/clavulanate | oxacillin resistance in S. aureus | r= -**0.9** | *p* = 0.007 |  |
|  |  |  |  | piperacillin/tazobactam | oxacillin resistance in S. aureus | r=-0.8 | *p* = 0.04, |  |
|  |  |  |  | Vancomycin | oxacillin resistance in S. aureus | r=-**0.8** | *P=0.04* |  |
|  |  |  |  | piperacillin/tazobactam | ESBL-positive Enterobacteriaceae | r=-0.8 | *p* = 0.01 |  |
|  |  |  |  | piperacillin/tazobactam | ESBL-positive *E. coli* | r= -**0.8** | *p* = 0.02 |  |
|  |  |  |  | piperacillin/tazobactam | Piperacillin/tazobactam resistant *Proteus* spp. | r=-**0.8** | **0.03** |  |
|  |  |  |  | Gentamicin | gentamicin resistant *E. coli* to gentamicin | r=+**0.8** | *p* = 0.03 |  |
|  |  |  |  | Gentamicin | *gentamicin resistant Klebsiella* spp | r=+**0.8** | *p* = 0.02 |  |
|  |  |  |  | carbapenems | carbapenem-resistant *A. baumannii* | r=+**0.8** | *p* = 0.03 |  |
|  |  |  |  | carbapenems | carbapenem-resistant MDR *A.baumannii* | r= +**0.7** | *p* = 0.05 |  |
|  |  |  |  | Cefepime | Cefepime *resistant A. baumannii* | **R=** +**0.9** | *P=0.01* |  |
| Correlation between antimicrobial consumption and antimicrobial resistance of Pseudomonas aeruginosa in a hospital setting: a 10-year study | Serbia, 2016 | *Retrospective* | Tertiary care university hospital | Imipenem | Carbapenems resistant P. aeruginosa | q = 0.758 | P < .005 | Mladenovic-Antic S., et al (34) |
|  |  |  |  | Meropenem |  | r = 0745 | P < 005 |  |
|  |  |  |  | carbapenems | Imipenem resistant P. aeruginosa | r = *0.795* | P < *001,* |  |
|  |  |  |  |  | Meropenem resistant P. aeruginosa | r = *0795* | P < *001,* |  |
|  |  |  |  | Amikacin | Aminoglycosides resistant P. aeruginosa | r == 0.661 | P < 005 |  |
|  |  |  |  | Gentamicin |  | r == 0.758 | P < 005 |  |
|  |  |  |  | Aminoglycoside | amikacin resistant P. aeruginosa | r = 0.837 | P < 001 |  |
|  |  |  |  |  | gentamicin resistant P. aeruginosa | r = 0.827 | P < 001 |  |
|  |  |  |  | all beta-lactam | imipenem resistnt P. aeruginosa | r = 0.847 | P < 001 |  |
|  |  |  |  | all beta-lactam | meropenem resiatant P. aeruginosa | r = 0.668 | P < 005 |  |
| Correlation between antimicrobial consumption and the prevalence of carbapenem‐resistant Escherichia coli and carbapenem‐resistant Klebsiella pneumoniae at a university hospital in Thailand | Thailand, 2018 | *Retrospective* | University Hospital | Fluoroquinolones | Carbapenem‐ resistant E. coli | r =-0.63 | 0.01 | Prakobsrikul N., et al (35) |
|  |  |  |  |  | Fluoroquinolones resistant *E. coli* |  |  |  |
|  |  |  |  |  | Carbapenem‐resistant K. pneumoniae | r =-0.60 | 0.02 |  |
|  |  |  |  |  | *Fluoroquinolones resistant K. pneumoniae* |  |  |  |
|  |  |  |  | Ceftriaxone | Carbapenem‐resistant | r =0.73 | <0.01 |  |
|  |  |  |  | Ciprofloxacin | *K. pneumoniae* | r =-0.73 | <0.01 |  |
|  |  |  |  | Levofloxacin | *K. pneumoniae* | r =0.65 | 0.01 |  |
|  |  |  |  | Carbapenem | *K. pneumoniae* | r =0.55 | 0.04 |  |
|  |  |  |  | Ertapenem | *K. pneumoniae* | r =0.54 | * 0.04 |  |
|  |  |  |  | Norfloxacin | Carbapenem‐resistant E. coli | r =0.54 | 0.04 |  |
|  |  |  |  |  | Carbapenem‐resistant K. pneumoniae | r =-0.82 | , <0.01 |  |
|  |  |  |  | Ofloxacin | Carbapenem‐resistant E. coli | r =-0.67 | 0.01 |  |
|  |  |  |  |  | Carbapenem‐resistant K.pneumoniae | r =-0.83 | <0.01 |  |
| Differences in antimicrobial consumption, prescribing and isolation rate of multidrug resistant *K. pneumoniae*, *P. aeruginosa* and *A. baumannii* on surgical and medical wards | Serbia, 2017 | *Retrospective* | Hospital setting | Ceftazidime, Cefepime, Imipenem | MDR K.pneumoniae |  | P<0.05 | **Zivanovic V., et al (36)** |
|  |  |  |  | Ceftazidime, Cefepime, Imipenem | MDR P. aeruginosa |  | P<0.05 |  |
|  |  |  |  | Ceftazidime, Cefepime, Imipenem | MDR *A*. *baumannii* |  | P<0.05 |  |
| Effects of various antimicrobial stewardship programs on antimicrobial usage and resistance among common gram-negative bacilli causing health care-associated infections: A multicenter comparison | Taiwan, 2016 | retrospective study | 3 hospitals | Carbapenem | Carbapenem-resistant A. baumannii | r =0.842, 0.96, 0.603 | P<0.05 | Lai C.-C., et al (37) |
|  |  |  |  | Ciprofloxacin | Ciprofloxacin-resistant P. aeruginosa | r =-0.922 |  |  |
|  |  |  |  |  | Ciprofloxacin-resistant A. baumannii | R=-0.666 |  |  |
|  |  |  |  | Piperacillin/tazobactam use | Piperacillin/tazobactam resistant A. baumannii, | R=0.866 ) |  |  |
|  |  |  |  | Carbapenem | Carbapenem-resistant P. aeruginosa. | r = 0.625 |  |  |
|  |  |  |  |  | Carbapenem-resistant K. pneumoniae | r = 0.665 |  |  |
|  |  |  |  | Extended-spectrum | Resistant P. aeruginosa | r =0.626 |  |  |
|  |  |  |  | cephalosporin | Resistant E. coli | r =0.739 |  |  |
|  |  |  |  | cephalosporin | Resistant A. baumannii, | r =0.817 |  |  |
| Emergence of linezolid-resistance in vancomycin-resistant *E.faecium* ST117 associated with increased linezolid-consumption | Germany, 2021 | Retrospective | A tertiary care hospital | Linezolid | vancomycin- resistant *E. faecium* | r = 0.63, | p = 0.017 | Olearo F., et al (38) |
| Five-year surveillance and correlation of antibiotic consumption and resistance of Gram-negative bacteria at an intensive care unit in Serbia | Serbia, 2020 | Retrospective | Hospital | Colistin, Tigecycline Fluoroquinolones | TZP resistant P. aeruginosa | r =- (0.53, 0.76, 0.79 ) |  | Popović R., et al (39) |
|  |  |  |  | Glycopeptides consumption | TZP resistant P. aeruginosa | R= 0.76 |  |  |
|  |  |  |  | 1st and 2nd gen. | TZP resistant P. aeruginosa | R=0.89 |  |  |
|  |  |  |  | Cephalosporins | TZP resistant P. aeruginosa |  |  |  |
|  |  |  |  | 3rd and 4th gen. | TZP resistant P. aeruginosa | R= 0.83 |  |  |
|  |  |  |  | Cephalosporins | TZP resistant P. aeruginosa |  |  |  |
|  |  |  |  | Aminoglycosides | TZP resistant P. aeruginosa | R=0.98 |  |  |
| Fluoroquinolone consumption and | Japan, 2019 | Retrospective |  | Fluoroquinolone | Levofloxacin-resistant E. coli | r = 0.52 | P< 0.01 | Terahara F.,et al. (40) |
| Escherichia coli resistance in Japan: an ecological study |  |  |  |  |  |  |  |  |
| Fluoroquinolone consumption and -resistance trends in Mycobacterium tuberculosis and other respiratory pathogens: Ecological antibiotic pressure and consequences in Pakistan, 2009–2015 | Pakistan, 2016 | Retrospective | Community and hospital | Fluoroquinolone | FQ-resistant MTB | R=0.2 | P<0.05 | Shakoor S., et al (41) |
|  |  |  |  |  | H. influenzae resistance rates | R=0.7009 | P<0.05 |  |
|  |  |  |  |  | Shigella spp. resistance rates | R=0.6898 | P<0.05 |  |
| Impact of antimicrobial stewardship managed by clinical pharmacists on antibiotic use and drug resistance in a Chinese hospital, 2010–2016: a retrospective observational study | China, 2019 | Retrospective | Hospital | imipenem/cilastatin | imipenem/cilastatin -resistant *E. coli* | r=0.8651 | p<0.05 | Wang H., et al (42) |
|  |  |  |  |  | Resistance rate of *K. pneumoniae* Imipenem/cilastatin | r=0.9050 | p<0.05 |  |
|  |  |  |  |  | imipenem/cilastatin resistant MRSA | r=-0.9611 | p<0.01 |  |
|  |  |  |  | Levofloxacin | levofloxacin resistant E. coli | r=0.8954 | p<0.05 |  |
|  |  |  |  | ciprofloxacin | ciprofloxacin-resistant E. coli | r=0.8950 | p<0.05 |  |
|  |  |  |  | ciprofloxacin | ciprofloxacin-resistant *K. pneumoniae* | r=0.9209 | p<0.01 |  |
|  |  |  |  | ciprofloxacin | ciprofloxacin resistant P. aeruginosa | r=0.8954 | p<0.05 |  |
|  |  |  |  | ciprofloxacin | ciprofloxacin resistant *P. aeruginosa* | r=0.9282 | p<0.01 |  |
|  |  |  |  | Levofloxacin | levofloxacin resistant MRSA | r=0.9450 | p<0.01 |  |
|  |  |  |  | ciprofloxacin | ciprofloxacin resistant MRSA | r=0.8883 | p<0.05 |  |
| Influence of antibiotic pressure on multidrug resistant Klebsiella pneumoniae colonization in critically ill patients | Spain, 2019 | A prospective observational | ICU in a tertiary hospital | cephalosporins | cephalosporins resistant K. pneumoniae | 0.616 | 0.013 | Ruiz J et al (43) |
|  |  |  |  | carbapenems | carbapenems resistant K. | 0.38 | 0.043 |  |
|  | Romania, 2017 | A prospective study | Tertiary care university hospital | Meropenem | carbapenem-resistant *P. aeruginosa* | r= 0.921 | p = 0.013 | Baditoiu L ., et al (44) |
|  |  |  |  | Carbapenems | Carbapenem-resistant *P. aeruginosa* | r=0.861 | p < 0.001 |  |
|  |  |  |  | β-lactam antibiotics with piperacillin/tazobactam | combined-resistant *P. aeruginosa* | r=0.698, | p = 0.010 |  |
|  |  |  |  | β-lactam antibiotics with piperacillin/tazobactam | combined-resistant *P. aeruginosa* | r=0.953, | p *=* 0.017 |  |
| Intra-hospital differences in antibiotic use correlate with antimicrobial resistance rate in Escherichia coli and Klebsiella pneumoniae: a retrospective observational study | Switzerland, 2018 | Retrospective | Tertiary care hospital | amoxicillin-clavulanic | Resistance rate in K. pneumoniae | OR=1.07 | p = 0.025 | Cusini A., et al (45) |
|  |  |  |  | Trimethoprim-sulfamethoxazole | Resistance rate in K. pneumoniae | OR= 2.02 | p < 0.001 |  |
|  |  |  |  | Amoxicillin-clavulanic acid | Amoxicillin-clavulanic acid resistant E.coli | OR=1.07 | P= 0.004 |  |
|  |  |  |  | Piperacillin-tazobactam | Piperacillin-tazobactam resistant E.coli | OR=2.11 | P<0.001 |  |
|  |  |  |  | Quinolone | Quinolone resistant E.coli | OR=1.52 | P<0.001 |  |
|  |  |  |  | Trimethoprim-sulfamethoxazole | Trimethoprim-sulfamethoxazole esistant E.coli | OR=1.59 | P<0.002 |  |
| Is There an Association Between Use of Amoxicillin-Clavulanate and Resistance to Third-Generation Cephalosporins Escherichia coli at the Hospital Level? | France, 2018 | Retrospective | Hospitals | 3GC | 3GC resistant K. pneumoniae | IRR=1.036 | P<0.0001 | Marquet A., et al (46) |
|  |  |  |  | Amoxicillin-clavulanate use | Amoxicillin-clavulanate resistant K. pneumoniae | IRR=-0.992 | p<0.001) |  |
|  |  |  |  | fluoroquinolones | fluoroquinolones resistant K. pneumoniae | IRR=1.013 | 0.03 |  |
|  |  |  |  | 3GCcephalosporins | 3GC resistant E.coli | IRR=1.009 | P<0.01 |  |
|  |  |  |  | Amoxicillin-clavulanate use | 3GC resistant K. pneumoniae | IRR=0.992 | P<0.02 |  |
| Lack of correlation between reduced outpatient consumption of macrolides and macrolide resistance of invasive Streptococcus pneumoniae isolates in Slovenia during 1997–2017 | Slovenia, 2017 | Retrospective | Ambulatory care | macrolides | erythromycin resistant S. pneumoniae | r = –0.20; | P = 0.408 | Kastrin T., et al (47) |
| National disparities in the relationship between antimicrobial resistance and antimicrobial consumption in Europe: an observational study in 29 countries | 29 countries in the European, 2017 | Retrospective | Community and hospital | fluoroquinolones | fluoroquinolons resistant E. coli | R=0.84 | P <0.001 | McDonnell L., et al (48) |
|  |  |  |  | All antimicrobial | fluoroquinolons resistant E. coli | 0.56 | P <0.001 |  |
|  |  |  |  | Cloxacillin | MRSA | R=0.64 | P <0.001 |  |
|  |  |  |  | All antimicrobial |  | R=0.53 | P <0.001 |  |
|  |  |  |  | All antimicrobial | K. pneumoniae resistant to carbapenems | r=0.58 | P <0.001 |  |
|  |  |  |  | Macrolide | S. pneumoniae resistant to macrolides | r=0.56 | P <0.001 |  |
|  |  |  |  | Aminoglycosides | P. aeruginosa resistant to aminoglycosides | r=0.67 | P <0.001 |  |
|  |  |  |  | Fluoroquinolones | K. pneumoniae resistant to fluoroquinolones | r=0.52 | P <0.001 |  |
|  |  |  |  | Gentamicin | E. faecalis resistant to high-level gentamicin | r=0.56 | P <0.001 |  |
| Population level consumption of cephalosporins and macrolides may select for reduced antimicrobial susceptibility to unrelated antimicrobials in Neisseria gonorrhoeae: an ecological analysis | 24 countries in the Europe, 2020 | Retrospective | Population level | Macrolide | Cefixime resistance *N. gonorrhoeae* | OR=2.3 | P= 0.038 | Kenyon C., et al (49) |
|  |  |  |  | Cephalosporin | Ciprofloxacin resistance *N. gonorrhoeae* | OR=2.7 | P=0.001 |  |
|  |  |  |  | Macrolide |  | OR= 13.2 | P<0.001 |  |
|  |  |  |  | Quinolone |  | OR=16.8 | 0.006 |  |
| Population-level macrolide consumption is associated with clarithromycin resistance in H. pylori: An ecological analysis | 52 countries, 2019 | Retrospective | general population | Macrolide | clarithromycin resistance in H. pylori | OR= 0.49 | P = 0.007 | Kenyon C. (50) |
|  |  |  |  |  |  |  |  |  |
| Prevalence of macrolide resistance in Treponema associated with macrolide consumption | Global, 2019 | Retrospective | population-level | Macrolide | Macrolide resistance T. pallidum | r= 0.7 | P=0.009 | Kenyon C. (51) |
|  |  |  |  |  |  |  |  |  |
| Previous Antibiotic Exposure and Antimicrobial Resistance Patterns of *Acinetobacter* spp. and *Pseudomonas aeruginosa* Isolated from Patients with Nosocomial Infections | Serbia, 2017 | Retrospective | tertiary healthcare institution | Aminoglycosides | resistant *Acinetobacter* spp | r=0.821 | p=0.023 | Djordjevic Z.M., et al (52) |
|  |  |  |  | Aminoglycosides |  | r=-0.857 | p=0.014 |  |
|  |  |  |  | Tigecycline |  | r=0.955 | p=0.001 |  |
|  |  |  |  | Aminoglycosides |  | r=-0.856 | p=0.014 |  |
| *P. aeruginosa* in the ICU: prevalence, resistance profile, and antimicrobial consumption | Brasil, 2018 | Retrospective | ICU | Amikicin | *resistant P. aeruginosa* | β=-0.361 | p=0.012 | **Ribeiro ACS et al(53)** |
|  |  |  |  | imipenem |  | β=-0.316 | p=0.029 |  |
|  |  |  |  | Meropenem |  | β=0.327 | p=0.023 |  |
|  |  |  |  | polymyxin |  | β=0.351 | p=0.014 |  |
| Temporal association between antibiotic use and resistance in Klebsiella pneumoniae at a tertiary care hospital | South Korea, 2018 | Retrospective | tertiary care hospital | β-lactam/β-lactamase inhibitors | Piperacillin/tazobactam -resistant K pneumoniae | R= 0.66 | p < 0.01 | Ryu S., et al (54) |
|  |  |  |  |  | Levofloxacin -resistant K pneumoniae | R=-0.60 | p = 0.01 |  |
|  |  |  |  | Third-generation cephalosporin | Piperacillin/tazobactam -resistant K pneumoniae | R=-0.11 | p = 0.65 |  |
|  |  |  |  |  | Ceftazidime -resistant K pneumoniae | R=0.64 | p < 0.01 |  |
|  |  |  |  |  | Levofloxacin -resistant K pneumoniae | R=0.50 | p = 0.03 |  |
|  |  |  |  | Fluoroquinolones | Piperacillin/tazobactam -resistant K pneumoniae | R=0.26 | p = 0.29 |  |
|  |  |  |  |  | Ceftazidime -resistant K pneumoniae | R=0.14 | p < 0.01 |  |
|  |  |  |  |  | Levofloxacin resistant K pneumoniae | 0.23 | p = 0.5 |  |
| Temporal Variation in Antibiotic Resistance of *Acinetobacter baumannii* in a Teaching Hospital in Tunisia: Correlation with Antimicrobial Consumption | Tunisa, 2019 | Retrospective | a tertiary care hospital | Imipinem | Imipinem resistance *Acinetobacter baumannii* | r =0.778 | *P* < 0.05 | Nadia J., Et al (55) |
|  |  |  |  |  |  |  |  |  |
|  |  |  |  | Fosfomycin | Fosfomycin resistance *Acinetobacter baumannii* | r = 0.558 | P=0.031 |  |
| Tetracycline use in the community may promote decreased susceptibility to quinolones in Escherichia coli isolates | France, 2017 | retrospective | Community | Tetracycline | quinolones resistant E. coli |  | 0.023 | Batard E., et al (56) |
|  |  |  |  | Tetracycline | nalidixic acid resistant E. coli |  | 0.04 |  |
| The Correlation between Defined Daily Dose/1000 Patient-day of Antimicrobials and the Resistance Rate of P. aeruginosa and A. baumannii: The Correlation between Defined Daily Dose/1000 Patient-day of Antimicrobials | Thailand, 2016 | Retrospective | Hospital | Amikacin | multi-drug resistant *P. aeruginosa* | r = -0.90 | *P* = 0.037 | Hongchumpae O., et al, (57) |
|  |  |  |  | Amikacin | Rates of multi-drug resistant strains of *P. aeruginosa* | r = 0.90 | *P* =0.041 |  |
|  |  |  |  | Amikacin | imipenem resistant P. aeruginosa | r = 0.89, | *P* = 0.042 |  |
|  |  |  |  | Imipenem | imipenem resistant *P.aeruginosa* | r = 0.96 | *P* = 0.011 |  |
|  |  |  |  | Ertapenem | multi-drug esistant P.aeruginosa | r = -0.90 | *P* = 0.037 |  |
|  |  |  |  | amikacin | multi-drug resistant *A. baumannii* | r = -0.95 | *P* = 0.014 |  |
|  |  |  |  |  | imipenem resistant *A. baumannii* | r = -0.95 | *P* = 0.013 |  |
|  |  |  |  |  | meropenem resistant *A. baumannii* | r = -0.94 | *P* =0.018 |  |
| The Impact of Antibiotic Consumption on Development of Acinetobacter Baumannii Resistance | Bosnia, 2016 | Retrospective | University hospital | gentamicin. | gentamicin of resistant Acinetobacter baumannii | R= 0.601 | p = 0.023 | Granov D., et al (58). |
| Trend and seasonality of community-acquired Escherichia coli antimicrobial resistance and its dynamic relationship with antimicrobial use assessed by ARIMA models | Spain, 2017 | Retrospective time series | Primary health care | Amoxicillin–clavulanic acid | Moxicillin-clavulanic resistant E. coli | 1.92% | 0.015 | Asencio Egea MÁ, et al (59) |
|  |  |  |  | Co-trimoxazole | Resistance rate of E. coli for co-trimoxazole | 24.40% | 0.038 |  |
|  |  |  |  | Fosfomycin | Resistance rate of E. coli for Fosfomycin | 24.50% | 0.024 |  |
| Trends and correlation between antibacterial consumption and carbapenem resistance in gram-negative bacteria in a tertiary hospital in China from 2012 to 2019 | China, 2021 | Retrospective | Teaching hospital | carbapenems | Resistance to carbapenems in A. baumannii |  | P<0.05 | Liang C., et al (60) |
|  |  |  |  |  | Resistance to carbapenems in Escherichia coli |  | P<0.05 |  |
|  |  |  |  | Cephalosporin/β-lactamase inhibitor | Resistance rate of A. baumannii to carbapenems |  | P < 0.01 |  |
|  |  |  |  | Tetracyclines | Resistance rate of A. baumannii to carbapenems |  | P < 0.05 |  |
|  |  |  |  | Use of quinolones in DDD per 1000 PDs | resistance rate of Burkholderia cepacia (B. cepacia) to carbapenems |  | P < 0.05 |  |
|  |  |  |  | Uses of carbapenems |  |  | P < 0.01 |  |
|  |  |  |  | Use of penicillin/β-Lactamase inhibitor (P/BLI) | Resistance of Enterobacter cloacae to carbapenems |  | P < 0.01 |  |
| Trends and correlation between antibiotic usage and resistance pattern among hospitalized patients at university hospitals in Korea, 2004 to 2012 | Korea, 2018 | Retrospective | University Hospitals | Aminoglycosides | Resistance E. coli to 3rd CEPs | R=-0.698 | P<.001 | Kim B., et al(61) |
|  |  |  |  | Carbapenems |  | R=0.828 | P<.001 |  |
|  |  |  |  | FQs |  | 0.556 | <.001 |  |
|  |  |  |  | Aminoglycosides | Resistance E. coli to Ciprofloxacin | -0.56 | <.001 |  |
|  |  |  |  | Carbapenems |  | -0.597 | <.001 |  |
|  |  |  |  | FQs |  | 0.547 | <.001 |  |
|  |  |  |  | Aminoglycosides | Resistance K. pneumoniae to 3rd CEPs | -0.521 | .<001 |  |
|  |  |  |  | Carbapenems |  | 0.569 | <.001 |  |
|  |  |  |  | FQs |  | 0.41 | 0.011 |  |
|  |  |  |  | FQs | Resistance K.pneumoniae toCiprofloxacin | 0.587 | <.001 |  |
|  |  |  |  | FQs | A. baumanii Ciprofloxacin | 0.379 | 23 |  |
|  |  |  |  | Carbapenems | A. baumanii to Imipenem | 0.896 | <.001 |  |
|  |  |  |  | FQs | P. aeruginosa to Ciprofloxacin | -0.367 | 0.028 |  |
|  |  |  |  | AGs | P. aeruginosa to Gentamicin | 0.78 | <.001 |  |
|  |  |  |  | Glycopeptides | S.aureus to Oxacillin | 0.376 | 24 |  |
| Trends and correlation of antibiotic susceptibility and antibiotic consumption at a large teaching hospital in China (2007–2016): a surveillance study. | China, 2019 | Retrospective | University Hospital | 4GC | Cefepime resistant K. pneumoniae | R=-0.763 | 0.01 | Wang R., et al (62) |
|  |  |  |  | Fluoroquinolone | Ciprofloxacin resistant K. pneumoniae | R=-0.849 | 0.002 |  |
|  |  |  |  | 4GC | Cefepime resistant E. coli | -0.763 | 0.01 |  |
|  |  |  |  |  |  |  |  |  |
|  |  |  |  | Fluoroquinolone | Ciprofloxacin resistant E. coli | -0.857 | 0.002 |  |
|  |  |  |  | Glycopeptides | Amikacin restant E. coli | -0.784 | 0.007 |  |
|  |  |  |  | Fluoroquinolone | Ciprofloxacin resistant A. baumannii | 0.772 | 0.015 |  |
|  |  |  |  | Glycopeptides | A. baumannii Amikacin | -0.871 | 0.001 |  |
|  |  |  |  | Fluoroquinolone | S. aureus Ciprofloxacin | -0.867 | 0.001 |  |
|  |  |  |  | Glycopeptides | S. aureus to Gentamicin | -0.818 | 0.004 |  |
|  |  |  |  | Fluoroquinolone | CoN staphylococcus Ciprofloxacin | -0.686 | 0.028 |  |
|  |  |  |  | Glycopeptides | CoN staphylococcus Gentamicin | -0.886 | <0.001 |  |
|  |  |  |  | Glycopeptides | E. faecium Amikacin | -0.827 | 0.003 |  |
|  |  |  |  | Fluoroquinolone | E. faecalis Ciprofloxacin | -0.968 | <0.001 |  |
|  |  |  |  | Glycopeptides | E. faecalis Gentamicin | -0.8 | 0.005 |  |
|  |  |  |  | Glycopeptides | Pseudomonas aeruginosa amikicin | −0.861 | 0.001 |  |
|  |  |  |  | Fluoroquinolone | Pseudomonas aeruginosa cipro | -0.647 | 0.043 |  |
| Trends and relationship between antimicrobial resistance and antibiotic use in Xinjiang Uyghur Autonomous Region, China: Based on a 3 year surveillance data, 2014–2016 | China, 2018 | A retrospective | 36 Hospitals | Beta-lactam–beta-lactamase inhibitor | of third-generation cephalosporin-resistant K. pneumonia | cc = 0.63 | p = 0.03 | Wushouer H., et al (63) |
|  |  |  |  | Quinolones |  | cc = 0.60 | p = 0.04 |  |
|  |  |  |  | Carbapenems |  | cc = 0.76, | p = 0.004 |  |
|  |  |  |  | Beta-lactam–beta-lactamase inhibitor | third-generation cephalosporin-resistant E. coli | cc = 0.80 | p = 0.003 |  |
|  |  |  |  | Carbapenem | carbapenem-resistant P. aeruginosa | cc = 0.65 | p = 0.02 |  |
|  |  |  |  | Penicillin |  | cc = 0.76 | p = 0.004 |  |
|  |  |  |  | Quinolone |  | cc = 0.69 | p = 0.01 |  |
|  |  |  |  | 3GC |  | 0.68 | < 0.05 |  |
|  |  |  |  | 3GC | MRSA | cc = 0.61 | p = 0.003 |  |
|  |  |  |  | Carbapenems |  | cc = 0.58 | p = 0.049 |  |
|  |  |  |  |  |  |  |  |  |
|  |  |  |  | Glycopeptides |  | cc = 0.63 | p = 0.03 |  |
| Trends in and correlations between antibiotic consumption and resistance of Staphylococcus aureus at a tertiary hospital in China before and after introduction of an antimicrobial stewardship programme | China, 2017 | Retrospective | Tertiary Hospitals | Monobactams | resistance to MRSA | 3.8 | 0 | Zhang D.,et al (64) |
|  |  |  |  | Glycopeptides |  | -2.83 | 0.005 |  |
|  |  |  |  | Oxazolidinone |  | -5.38 | 0 |  |
|  |  |  |  | Aminoglycosides |  | -3.06 | 0.002 |  |
|  |  |  |  | Imidazole |  | 6.41 | 0 |  |
|  |  |  |  | Sulphonamides |  | 4.01 | 0 |  |
| Trends in South Korean antimicrobial use and association with changes in *Escherichia coli* resistance rates: 12-year ecological study using a nationwide surveillance and antimicrobial prescription database. | S.Korea (2018) | Retrospective | Hospitals | Fluoroquinolone | resistance rates to fluoroquinolone in *E. coli* | r = 0.82 | *P* = 0.0012 | Kim Y.A., et al (65) |
|  |  |  |  | Ciprofloxacin |  | r = 0.90, | *P<*0.0001 |  |
|  |  |  |  | Third-generation cephalosporin use | Resistance rate to cefotaxime in *E.coli* | r = 0.96,) | *P<*0.0001 |  |
|  |  |  |  | Cefotaxime use |  | r = 0.94 | *P<*0.0001 |  |
|  |  |  |  | Third- and fourth-generation cephalosporin use |  | r = 0.99 | *P<*0.0001 |  |
|  |  |  |  | **Cephamycin use** | **Resistance rates to cefoxitin in** | r = 0.64, | 0.0256 |  |
|  |  |  |  | Cefoxitin use | ***E.coli*** | r = 0.73 | *P* = 0.0158 |  |
| Trends, seasonality and the association between outpatient antibiotic use and antimicrobial resistance among urinary bacteria in the Netherlands | Netherlands, 2020 | Retrospective | Hospital | Nitrofurantoin | Nitrofurantoin resistance rate for in E. coli | F = 2.52 | P = 0.05 | Martı ´nez E.P., et al (66) |
|  |  |  |  | Ciprofloxacin | Ciprofloxacin in K. pneumoniae. | F = 3.86 | P = 0.01 |  |
|  |  |  |  | Trimethoprim | Resistance to trimethoprim in K. pneumoniae | F = 4.56 | P = 0.002 |  |
| **Evaluating the Relationship Between Hospital Antibiotic Use and Antibiotic Resistance in Common Nosocomial Pathogens** | Canada, 2017 | observational ecological study | academic and community hospitals | All antibiotic | Escherichia coli |  | 0.005 | Wang A., et al (67) |
|  |  |  |  |  | K pneumonia |  | 0.004 |  |
|  |  |  |  |  | Enterobacter spp |  | 0.003 |  |
|  |  |  |  |  | Enterococcus spp |  | 0.001 |  |
| A Nonlinear Time-Series Analysis to Identify the Thresholds in Relationships Between Antimicrobial Consumption and Resistance in a Chinese Tertiary Hospital | China, 2022 | retrospective | Tertiary Hospital | glycopeptides | Carbapenem-resistant Klebsiella pneumoniae | 0.652 |  | Chen S., et al. (68) |
|  |  |  |  | Carbapenem |  | 0.738 |  |  |
|  |  |  |  | glycopeptides | Carbapenem-resistant Acinetobacter baumannii | R2 = 0.644 |  |  |
|  |  |  |  | carbapenems |  | R2 = 0.647 |  |  |
|  |  |  |  | carbapenems | Aminoglycosides-Resistant Pseudomonas aeruginosa | R2 = 0.632 |  |  |
|  |  |  |  | glycopeptides |  | R2 = 0.348 |  |  |
|  |  |  |  | fluoroquinolones | Carbapenem-resistant Escherichia coli | 0.49 |  |  |
|  |  |  |  | carbapenems |  | R2 = 0.387 |  |  |
| Antibiotic Consumption and Its Relationship with Bacterial Resistance Profiles in ESKAPE Pathogens in a Peruvian Hospital | Peru, 2021 | prospective | Hospital, Wards | piperacillin/tazobactam | piperacillin/tazobactam resistant Pseudomonas aeruginosa | 0.95 | 0.051 | Pérez-Lazo G., et al (69) |
|  |  |  |  |  | *piperacillin/tazobactam resistant Enterobacter* spp | 0.95 | 0.051 |  |
|  |  |  |  |  | imipenem resistant K Pneumonia | 0.95 | 0.051 |  |
|  |  |  |  |  | meropenem resistant K Pneumonia | 0.95 | 0.051 |  |
|  |  |  |  | meropenem | piperacillin/tazobactam resistant Enterobacter spp | 0.95 | 0.051 |  |
|  |  |  |  |  | meropenem resistant Pseudomonas aeruginosa | 0.95 | 0.051 |  |
|  |  |  |  | ertapenem | imipenem resistant Pseudomonas aeruginosa | 0.95 | 0.051 |  |
|  |  |  |  | ceftazidime | meropenem resistant Acinetobacter baumannii | 0.93 | 0.003 |  |
|  |  |  |  |  | imipenem resistant Acinetobacter baumannii | 0.93 | 0.003 |  |
|  |  |  |  |  | meropenem resistant Pseudomonas aeruginosa | 0.93 | 0.003 |  |
|  |  |  |  | ciprofloxacin | *piperacillin/tazobactam resistant Enterobacter* spp | r2=-0.97 | 0.031 |  |
|  |  |  |  |  | imipenem resistant Acinetobacter baumannii | r2=-0.89 | 0.41 |  |
|  |  |  |  |  | meropenem resistant Acinetobacter baumannii | r2=-0.89 | 0.41 |  |
|  |  |  |  | imipenem | imipenem resistant K Pneumonia | r2=-.95 | 0.051 |  |
|  |  |  |  |  | meropenem resistant K Pneumonia | r2=-.96 | 0.051 |  |
|  |  |  |  | Clindamycine | OXA resistant S aurous | **r=** *-*0.94 | 0.051 |  |
|  |  |  |  | Vancomycine | Vancomycine resistant Enterococcus faecium | 0.95 | 0.051 |  |
| Antimicrobial resistance and antibiotic consumption in intensive care units, Switzerland, 2009 to 2018 | Switzerland, 2021 | Retrospective | Hospital ICU | extended-spectrum cephalosporin | Methiciline- resistant Staphylococcus aureus | R2 = 0.87 | P< 0.001 | Barnsteiner S., et al (70) |
|  |  |  |  | extended-spectrum cephalosporin | extended-spectrum cephalosporin-resistant Escherichia coli | R2 = 0.84 | p < 0.001 |  |
|  |  |  |  | carbapenem | carbapenem-resistant Enterobacterales | R2 = 0.56 | p = 0.008 |  |
|  |  |  |  | extended-spectrum cephalosporin | extended-spectrum cephalosporin-resistant Klebsiella pneumoniae | R2 = 0.84 | p < 0.001 |  |
|  |  |  |  | glycopeptid | glycopeptid-resistant Enterococcus faecalis/faecium | R2 = 0.52 | p = 0.011 |  |
| Correlation between Antibiotic Consumption and Resistance of Invasive Streptococcus pneumoniae | Slovenia, 2021 | Retrospective | Hospital | all antibiotic | resistance of *S. pneumoniae* | 0.84 | 0.001 | Cižman M., et al. (71) |
|  |  |  |  | penicillins |  | 0.83 | 0.001 |  |
|  |  |  |  | extended spectrum penicillin and penicillin with β-lactamase inhibitors |  | 0.64 | 0.001 |  |
| Correlation between antibiotic consumption and resistance of Pseudomonas aeruginosa in a teaching hospital implementing an antimicrobial stewardship program: A longitudinal observational study | China, 2022 | Longitudinal observational study | Hospital | Piperacillin/ tazobactam | P. aeruginosa to respective antibiotics | 0.221 | 0.012 | Huang H-W., et al (72) |
|  |  |  |  | Ceftazidime |  | 0.191 | 0.019 |  |
|  |  |  |  | Cefepime |  | 0.112 | 0.028 |  |
|  |  |  |  | Imipenem/cilastatin |  | 0.132 | 0.051 |  |
|  |  |  |  | Gentamicin |  | 0.573 | <0.0001 |  |
|  |  |  |  | Amikacin |  | 0.487 | <0.0001 |  |
|  |  |  |  | Ciprofloxacin |  | 0.135 | 0.055 |  |
|  |  |  |  | Levofloxacin |  | 0.517 | <0.0001 |  |
| Correlation Between Individual Child-Level Antibiotic Consumption and Antibiotic-Resistant Among Commensal Escherichia coli: Results from a Cohort of Children Aged 1–3 Years in Rural Ujjain India | India, 2022 | Prospective Cohort | Community | cephalosporins | cefotaxime resistance E. coli |  | 0.004 | Khare S., et al (73) |
| Country-level association between antimicrobial consumption and resistance in Neisseria meningitidis: An ecological study | 13 European countries, 2022 | ecological study | Community | quinolones | ciprofloxacin resistance to Neisseria meningitidis |  | 0.003 | Manoharan-Basil S.S., et al (74) |
|  |  |  |  | cefotaxime | efotaxime resistance to Neisseria meningitidis |  | 0.001 |  |
| Helicobacter pylori resistance to antibiotics in Europe in 2018 and its relationship to antibiotic consumption in the community | Europe, 2021 | observational | community | macrolides | Clarithromycin resistant in H. pylori |  | 0.0019 | Megraud F., et al (75) |
|  |  |  |  | quinolones | Levofloxacin resistant in H. pylori |  | 0.0002 |  |
| Identification of thresholds in relationships between specific antibiotic use and carbapenem-resistant Acinetobacter baumannii (CRAb) incidence rates in hospitalized patients in Jordan | Jordan, 2021 | retrospectively | University Hospital | 3GC | carbapenem-resistant Acinetobacter baumannii | r2>0.394 | <0.001 | Hayajneh W.A., et al (76) |
|  |  |  |  | Carbapenems | carbapenem-resistant Acinetobacter baumannii | r2>0.395 | 0.0139 |  |
| Trends and Correlation Between Antimicrobial Resistance and Antibiotics Consumption in a Specialist Children’s Hospital from 2016 to 2021 | China, 2022 | retrospective | Children Hospital | Cephalosporine/BLI | E. cloacae to carbapenems | 0.675 | <0.001 |  |
|  |  |  |  | Penicillin/BLI | E. cloacae to carbapenems | 0.684 | <0.001 |  |
|  |  |  |  | Carbapenems | E. cloacae to carbapenems | 0.417 | 0.043 |  |
|  |  |  |  | Monobactams | E. cloacae to carbapenems | 0.507 | 0.011 |  |
|  |  |  |  | Cephalosporine/BLI | *A. baumannii* to carbapenems | 0.763 | <0.001 |  |
|  |  |  |  | Penicillin/BLI | *A. baumannii* to carbapenems | 0.77 | <0.002 |  |
|  |  |  |  | Cephalosporine/BLI | *P. aeruginosa* to ceftazidime | *0.625* | 0.001 |  |
|  |  |  |  | Penicillin/BLI | *P. aeruginosa* to ceftazidime | 0.632 | 0.001 |  |
|  |  |  |  | carbapenems | carbapenem resistant A. baumannii | 0.806 | 0.001 |  |
|  |  |  |  |  | carbapenems resistant E. cloacae | 0.417 | 0.043 |  |
|  |  |  |  |  | ceftazidime resistant P. aeruginosa | 0.753 | <0.001 |  |
|  |  |  |  | monobactams | resistance rates of A. baumannii to carbapenems | 0.557 | 0.005 |  |
|  |  |  |  |  | carbapenems resistant E. cloacae | 0.0597 | 0.011 |  |
|  |  |  |  | Cephalosporine/BLI | ceftazidime resistant P. aeruginosa | 0.625 | 0.001 |  |
|  |  |  |  | B-lactam/BLI | ceftazidime resistant P. aeruginosa | 0.632 | 0.001 |  |
|  |  |  |  | Cephalosporins | A.baumannii to carbapenems | 0.401 | 0.053 |  |
|  |  |  |  | Cephalosporine/BLI | A.baumannii to carbapenems | 0.763 | <0.001 |  |
|  |  |  |  | B-lactam/BLI | A.baumannii to carbapenems | 0.77 | <0.001 |  |
|  |  |  |  | Carbapenems | A.baumannii to carbapenems | 0.806 | <0.001 |  |
